# Priorities for the development of a new rapid diagnostic test for patients with fever: a cross-sectional online survey among hospital physicians across Europe

**DOI:** 10.1136/bmjopen-2025-107663

**Published:** 2026-03-24

**Authors:** Gabrielle Bonnet, Maryke Joanne Nielsen, Anna M Foss, Alex Lewin, Ruud Gerard Nijman, Elizabeth Fitchett, Enitan Carrol, Shunmay Yeung, the DIAMONDS consortium, Gabrielle Bonnet

**Affiliations:** 1 London School of Hygiene and Tropical Medicine, London, UK; 2 Alder Hey Children’s NHS Foundation Trust, Liverpool, England, UK; 3 Public Health and Policy, London School of Hygiene and Tropical Medicine, London, England, UK; 4 Imperial College Healthcare NHS Trust, London, England, UK; 5 Institut Pasteur de Dakar, Dakar, Dakar Region, Senegal; 6 University of Liverpool, Liverpool, UK; 7 Alder Hey Children’s Hospital, Liverpool, England, UK; 8 St Mary’s Imperial College Hospital, London, England, UK

**Keywords:** Clinical Decision-Making, Emergency Departments, Molecular diagnostics, Paediatric A&E and ambulatory care, PUBLIC HEALTH, Surveys and Questionnaires

## Abstract

**Abstract:**

**Objective:**

This study aimed to understand hospital doctors’ priorities (target use cases and aetiologies) for the development of a new rapid diagnostic test for patients with fever.

**Design:**

A cross-sectional online survey.

**Setting:**

Europe-wide.

**Participants:**

Secondary and tertiary care doctors involved in patient assessment and diagnosis across Europe.

**Intervention:**

Online survey from April to September 2024.

**Main outcome measures:**

Importance of developing a new test on a scale of 1–10 for up to 19 ‘use cases’ (types of febrile presentations in specific demographic groups): use case scores and ranks and differences across subgroups of respondents, with free text to capture additional suggestions; respondents’ preferences (multiple choice) regarding which aetiologies should be included in a new test.

**Results:**

265 respondents from 30 European countries (out of 270 starting the survey) were included in the analysis. Top priorities included febrile immunocompromised patients and fever without a focus for both paediatric and adult use cases, and 1–3 months old febrile infants. Rankings were similar across clinician subgroups despite some differences in average scores. 92% (243/263), 95% CI 89% to 95%, of respondents would find a ‘generic’ test for bacterial aetiology useful, even if it does not differentiate between Gram-positive and Gram-negative aetiologies. 54% (63/116), 95% CI 45% to 63%, of respondents would find a ‘generic’ test for inflammatory aetiology useful when seeking to diagnose children for whom Kawasaki’s disease (KD) is on the differential, even in the absence of any KD-specific test, 83% (96/116), 95% CI 75% to 89%, would find such a ‘generic’ test useful if they could use it alongside a KD test when desired.

**Conclusion:**

Clinicians prioritise the most vulnerable patients (because of age or comorbidities) and unclear presentations (fever without a focus) for the development of a new fever diagnostic test. Even relatively simple (eg, bacterial, inflammatory) tests could provide added value to most clinicians.

**STRENGTHS AND LIMITATIONS OF THIS STUDY**

- This survey fills a critical research gap concerning Europe’s hospital clinicians’ priorities and preferences.
- Respondents cover a broad range of 30 European countries, including all large countries except Russia and Ukraine.
- Extensive descriptive and statistical analyses and subgroup analyses help ensure the robustness of the results.
- Dissemination through professional societies and research networks helped reach the target population but may nevertheless have contributed to over-representation of some respondent subgroups.
- Doctors willing to respond to the survey may have differed from non-respondents, for example, in their preference for online tools or their mastery of the English language.

## Introduction

 Clinicians regularly see patients presenting with suspected infection whose aetiology is uncertain when making decisions to treat, admit, discharge or send to a higher level of care.^1^ Consequently, some patients with severe illness are missed, sometimes leading to disability or death, while others are unnecessarily hospitalised, undergo additional invasive or risky investigations, or are presumptively treated, for example, with antibiotics, contributing to antimicrobial resistance (AMR).^2 3^ All these have potentially high associated costs.^4^ The development of new tests, sufficiently rapid to inform early clinical decisions, could improve patients’ outcomes and reduce costs.

There has recently been rapid progress in the development of such tests.^5^,^9^ The European Union (EU)-funded DIAMONDS project (Diagnosis and Management of Febrile Illness using RNA Personalised Molecular Signature Diagnosis), in particular, is developing a new rapid blood biomarker-based test to differentiate between bacterial, viral and other important causes of febrile illness.^10 11^ However, there is often a gap between ‘discovery’, the identification of candidate markers in laboratories and their appearance in a test in clinicians’ hands. To be cost-effective, tests should be used and have an impact on clinical decisions. This is most likely if they are deemed useful by end users, and their implementation aligns with existing care pathways. However, while a previous survey has described rapid tests availability and use in paediatric settings in Europe,^12^ to our knowledge, few have identified clinicians’ test development preferences.

This study addresses this critical gap for European hospital settings.

## Materials and methods

### Study aims

This study aims to support the optimisation of new rapid biomarker-based multiplex test development through a better understanding of the perspectives of hospital clinicians responsible for diagnosing and managing patients presenting with febrile illness in Europe. Our primary objective was to understand in which ‘use cases’ (patient profile and clinical presentation) new tests would be most useful in aiding initial clinical decision-making. Secondary objectives included:

- Better understanding the relative value of generic versus more specific tests.
- Assessing differences in clinicians’ responses depending on their profiles.

### Study design

We collected cross-sectional data through an open online survey from 12 April to 27 September 2024^13^ .

### Target population

We targeted doctors with diverse experience levels, work settings, countries of work and specialties, working in hospitals in Europe and involved in the acute assessment and management of patients.

### Inclusion and exclusion criteria

Respondents were included if they were doctors working in a hospital in Europe (including non-EU countries) and involved in patient assessment and diagnosis. Exclusion criteria were: (1) not being a doctor (eg, an allied health professional), (2) not working in a hospital (eg, in general practice), (3) working outside of Europe and (4) not consenting to the survey.

### Survey development processes

The survey was developed through an iterative process involving the core study team and piloting with a wider group of 16 clinicians involved in the DIAMONDS consortium. To prioritise clinical need over specific technical specifications during the discovery phase of the test, the questionnaire asked respondents to consider ‘host biomarker-based’ and ‘multi-aetiology’ tests, without specifying the technical characteristics of the test and its cost.

### Sample size

Sample size calculations (a minimum of 186 respondents in total and 22 per subgroup for subgroup analyses) were based on answers to the pilot. Details are in online supplemental appendix S1.

### Survey tools

The survey is available in online supplemental appendix S2. It comprised three sections. The first section asked about respondents’ experience, trainee versus consultant status, work setting, country of practice, specialty and whether they managed neonates, children and/or adult patients.

In the second section, the new test was briefly described as a host biomarker-based multiplex test which could identify some combination of: bacterial, Gram-positive bacterial, Gram-negative bacterial, viral, Kawasaki’s disease (KD), multisystem inflammatory syndrome in children, other inflammatory aetiologies, tuberculosis and/or malaria. These aetiologies were based on prior discovery research from the DIAMONDS consortium.^10^ Depending on the age group(s) respondents reported managing, they were then presented with a list of use cases (table 1): neonatal (2), paediatric (11) and/or adult (6). Clinicians scored how important they felt each use case was as a target for the development of a new rapid test, from 1 (not important) to 10 (extremely important). A free text field allowed for additional suggestions.

**Table 1:** List of use cases per patient age group

Patient age group	Use case number	Description
Neonates	1a	‘Unwell neonate – early onset’ (< 3 days)—differential includes infection, ischaemic-hypoxic encephalopathy, congenital malformation, etc
Neonates	1b	‘Unwell neonate – later onset’ (3–28 days)—differential includes sepsis, congenital malformation, etc
Children	2a	Very young febrile infant (1–3 months)
Children	2b	Bronchiolitis (<18 months) with or without bacterial infection
Children	2c	Child for whom Kawasaki’s disease is on the differential
Children	2d	Unwell or deteriorating child with a fever without focus
Children	2e	Prolonged fever (>10 days)—unclear aetiology
Children	2f	Child with suspicion of meningoencephalitis
Children	2g	Suspected pneumonia
Children	2h	Possible appendicitis
Children	2i	Atraumatic limp or bone/joint focus with or without a fever
Children	3a	Febrile neutropenic child (and other immunocompromised children)
Children	3b	Child with sickle cell disease and fever
Adults	4a	Fever without a focus, possible sepsis
Adults	4b	Prolonged fever (>10 days)—unclear aetiology
Adults	4c	Fever and respiratory deterioration—possible infection, embolism or cardiac aetiology
Adults	4d	Immunocompromised adult with fever
Adults	4e	Atraumatic musculoskeletal problem with or without a fever
Adults	4f	Elderly patient with a sudden non-specific deterioration, for example, falls, confusion, lethargy

In the third section, respondents were asked whether a test for bacterial aetiology only distinguishing between bacterial and non-bacterial would be as useful as one also identifying whether the bacteria was Gram-positive or Gram-negative. A similar question was asked of clinicians working with children regarding their preference for a specific KD test, a generic test for inflammatory aetiology or both.

All data were collected anonymously using the Online Surveys platform V.3.

### Participant recruitment

The survey was disseminated through the newsletters and listservs of professional societies and networks including the European Society for Emergency Medicine’s ‘Research in European Paediatric Emergency Medicine’ network; the European Society for Paediatric Infectious Diseases; the European Society of Clinical Microbiology and Infectious Diseases and the European Society for Paediatric Research, the Hellenic Infectious Diseases and Internal Medicine Societies and the DIAMONDS research network.

### Analysis

We assessed (using R V.4.4.1) whether differences in ranking between use cases were significant using a Wilcoxon signed-rank test for the two neonatal use cases and Friedman tests for paediatric and adult use cases. Ties were assigned an average rank. This was followed by pairwise comparisons, computing the p values for the difference in ranks following Eisinga *et al*’s^14^ method with Benjamini-Hochberg adjustment to control for the false discovery rate.^15^

We analysed use case rankings within subgroups of clinicians defined a priori and where sample size was sufficient and compared rankings across subgroups. We focused on the acute care clinicians most often responsible for making initial clinical decisions with limited information; district/regional versus teaching/university hospitals; clinicians with less versus more experience; and over-represented subgroups (to understand their impact on results).

Clinicians may differ in the overall priority they attribute to testing across use cases. This may reflect individual scoring practices or different interest in new diagnostic tests. We compared scoring between subgroups using (1) multilevel ordered beta regression,^16^ which accounts for the metric nature of clinicians’ responses (ie, 1–10 scale) and (2) ordinal regression using a cumulative link mixed effects model,^17 18^ which treats the scale as ordinal but better accounts for its discrete nature.

Text responses to the open question were reviewed and grouped through thematic analysis^19^ to identify recurrent themes.

Finally, for the questions asking clinicians about the aetiologies included in the test, we calculated the percentage of clinicians selecting each possible answer and associated CIs, then analysed subgroup differences using Fisher’s exact test and ordinal regression.

### Reporting

This survey follows the Checklist for Reporting Results of Internet E-Surveys guidelines and is reported using the CROSS (A Consensus-Based Checklist for Reporting of Survey Studies) checklist (online supplemental appendix S4).^20^

### Patient and public involvement

Patients and/or the public were not involved in the design, or conduct, or reporting, or dissemination plans of this research.

## Results

### Respondents

**Figure 1:**
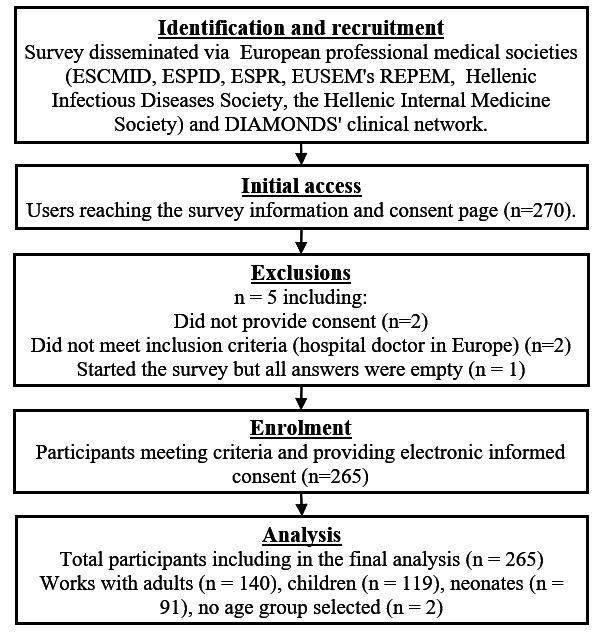
Flow chart showing the selection process of research participants. Numbers for clinical specialties sum to more than 265 as some respondents worked with both adult and paediatric age groups. DIAMONDS, Diagnosis and Management of Febrile Illness using RNA Personalised Molecular Signature Diagnosis; ESCMID, European Society of Clinical Microbiology and Infectious Diseases; ESPID, European Society for Paediatric Infectious Diseases; ESPR, European Society for Paediatric Research; EUSEM, European Society for Emergency Medicine; REPEM, Research in European Paediatric Emergency Medicine.

270 respondents reached the information and consent page and 265 respondents from 30 European countries were included in the analysis (table 2). This included 140, 119 and 91 working with adults, children and neonates, respectively; 81 worked with both neonates and children, while 6 worked with both adults and children, 3 of whom also worked with neonates. The participant selection process and attrition are illustrated in figure 1. Response rates for individual use cases are in online supplemental appendix S3.2.1.

**Table 2:** Respondents’ profile

	Number			%
Reached the information and consent page	**270**			**100.0**
Did not consent	2			0.7
Consented but not in target group	2			0.7
Started the survey but all answers were empty	1			0.4
Patient age group (multiple answers allowed)*	**Number**			**%**
Works with adults	140			52.8
Works with children	119			44.9
Works with neonates	91			34.3
None selected	2			0.8
Specialty (only one answer was allowed)	**Paediatric**	**Adult**	**Total**	**%**
Infectious disease/immunology	58	83	141	53.2
General or no specialty	21	16	37	14.0
Emergency medicine	25	1	26	9.8
Internal medicine	0	16	16	6.0
Neonatology	11	0	11	4.2
Intensive care/acute medicine	2	7	9	3.4
Diabetes/endocrinology	0	6	6	2.3
Haematology/oncology	2	2	4	1.5
Rheumatology	0	4	4	1.5
Gastroenterology	2	1	3	1.1
Respiratory medicine	2	0	2	0.8
Other specialties	3	2	5	1.9
No answer	0	1	1	0.4
**Total**	**126**	**139**	**265**	**100**
Setting (multiple answers allowed)	**Paediatric**	**Adult**	**Total**	**%**
Teaching/university hospital alone	102	77	179	67.5
District/regional hospital alone	18	38	56	21.1
Multiple or other	6	24	30	11.3
**Total**	**126**	**139**	**265**	**100**
Experience (ie, years worked as a clinician)	**Paediatric**	**Adult**	**Total**	**%**
1–2 years	6	3	9	3.4
3–4 years	10	5	15	5.7
5–10 years	23	23	46	17.4
11–20 years	40	57	97	36.6
>20 years	46	50	96	36.2
No answer	1	1	2	0.8
**Total**	**126**	**139**	**265**	**100**
Professional status	**Paediatric**	**Adult**	**Total**	**%**
Intern/trainee	15	15	30	11.3
Consultant/attending physician	99	112	211	79.6
Other	12	10	22	8.3
No answer	0	2	2	0.8
**Total**	**126**	**139**	**265**	**100**

* Groups are not mutually exclusive as some respondents work across multiple age groups.

Just over half of respondents were infectious disease doctors, while only one adult physician was an emergency medicine specialist. A large majority were consultants/attending physicians and two-thirds worked in teaching/university hospitals. There were similar shares of clinicians in the 10 years or less, 11–20 years and over 20 years of experience subgroups (the lower experience categories were merged). There was a fairly representative distribution of paediatric respondents across countries, but 74% of adult clinicians came from Greece (see online supplemental appendix S3). Finally, while 29% of respondents were aware of the DIAMONDS consortium through professional outreach, direct involvement was negligible (<2%). This small minority was included in the primary analysis, which reflects a broadly independent clinical consensus.

## Use case scoring and ranking

### Use case scoring

**Figure 2:**
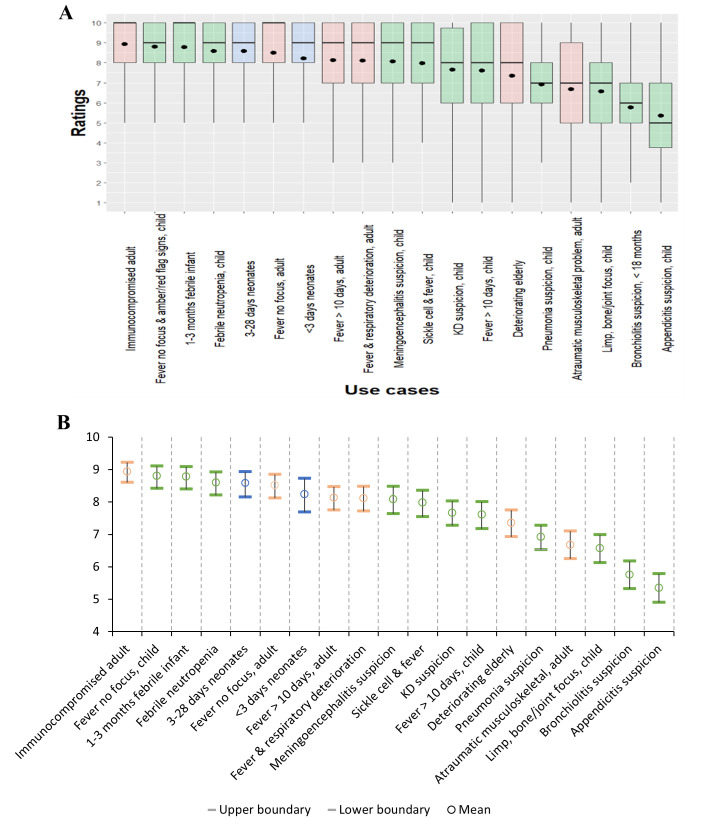
Clinician priority scores for 19 clinical use cases: (**A**) Distribution of respondent scores for each use case, represented as box-and-whisker plots. (**B**) Mean priority scores with associated 95% CIs. Use case age group is indicated by background shading: blue for neonatal, green for paediatric and pink for adults. In A, black dots represent the mean score, horizontal lines indicate the median, boxes represent the IQR and whiskers show the full data range. In B, error bars represent 95% CIs obtained through the adjusted bootstrap percentile method. Mean scores ranged from approximately 5.4–8.9, with the highest priorities attributed to immunocompromised adults; children with a fever without a focus (and amber/red flag signs); febrile infants (1–3 months); and children with febrile neutropenia. Use case descriptions on the x-axis are abbreviated; full clinical descriptions are provided in online supplemental appendix S1. Alt text: (**A**) Boxplot of the scores (mean, median, quartiles, range) of neonatal, paediatric and adult use cases. Alt text: (**B**) Mean scores and 95% CIs for neonatal, paediatric and adult use cases. KD, Kawasaki’s disease.

Figure 2 describes respondents’ mean scores for each use case using boxplots (Panel A) and confidence intervals (Panel B). Mean scores ranged from above five to just below 9. The four use cases with the highest mean scores are immunocompromised adults, children with a fever without a focus and amber/red flag signs, 1–3 months old febrile infants and febrile neutropenic children.

### Use case ranking

Friedman tests, applied to the subsets of complete paediatric and adult use case responses, showed that there are differences in ranking among both groups ($p§amp;lt;2.2\times 10^{-16}$ for both paediatric and adult use cases). The results of pairwise comparisons are presented in table 3, using the Compact Letter Display (CLD) approach^21^ (use cases are attributed a set of letters so that, when the difference in ranking between two use cases is not significant at the 5% level, they share a letter). The top three paediatric use cases (fever without a focus, 1–3 months old febrile infants and children with febrile neutropenia) were ranked similarly but higher than other paediatric use cases. Other highly ranked paediatric use cases were suspicion of meningoencephalitis, sickle cell disease and fever, prolonged fever and KD on the differential. Among adult use cases, the top two (immunocompromised adults and fever without a focus) are ranked similarly but are significantly higher ranked than other use cases. Unwell older neonates (3–28 days old) were ranked a little higher than younger neonates (<3 days old), but the difference was not significant (p=0.86, Wilcoxon signed-rank test). Pairwise comparisons across neonatal and paediatric use cases among clinicians involved in the diagnosis of both age groups suggest that neonatal use cases rank among the top five paediatric use cases. Additional details are in online supplemental appendix S3.2 and S3.3.

**Table 3:** Comparison of rankings between use cases using the CLD approach

Neonatal use cases	Average score*	CLD†
Unwell neonates, early onset (<3 days old)	8.6	a
Unwell neonates, late onset (3–28 days old)	8.2	a
**Paediatric use cases**	Average score*	**CLD†**
Unwell or deteriorating child with a fever without focus and amber/red flag signs	8.9	a
Very young febrile infant (1–3 months)	8.8	a
Febrile neutropenic child (and other immunocompromised children)	8.7	a b
Child with suspicion of meningoencephalitis	8.2	b c
Child with sickle cell disease and fever	8.0	c
Prolonged fever (>10 days)—unclear aetiology	7.6	c
Child for whom Kawasaki’s disease is on the differential	7.6	c
Suspected pneumonia	6.9	d
Atraumatic limp or bone/joint focus with or without a fever	6.5	d
Possible appendicitis	5.3	e
Bronchiolitis (<18 months) with or without bacterial infection	5.7	e
**Adult use cases**	Average score*	**CLD†**
Immunocompromised adult with fever	9.0	a
Fever without a focus, possible sepsis	8.6	a b
Fever and respiratory deterioration: possible infection, embolism or cardiac aetiology	8.2	b c
Prolonged fever (>10 days)—unclear aetiology	8.2	c
Elderly patient with a sudden non-specific deterioration, for example, falls, confusion, lethargy	7.4	d
Atraumatic musculoskeletal problem with or without a fever	6.7	e

* Clinicians with complete answers only: paediatric answers: n=106, adult answers: n=129. Use cases are ordered as per their average ranking.

† Use cases not sharing any letter have significantly different ranking at the 5% significance level: hence, the difference between a use case attributed the letters ‘ab’ and another with the letters ‘bcd’ would not be significant. The p values for the difference in ranks for paediatric and adult use cases were computed following Eisinga *et al*’s method,^14^ adjusted as per Benjamini-Hochberg.^15^

CLD, Compact Letter Display.

### Subgroup analyses

#### Analysis of ranking

Online supplemental appendix S3.4 shows rankings within subgroups in CLD notation and highlights the high level of agreement in rankings across subgroups for adult, paediatric and neonatal use cases.

#### Differences in average scores

**Figure 3:**
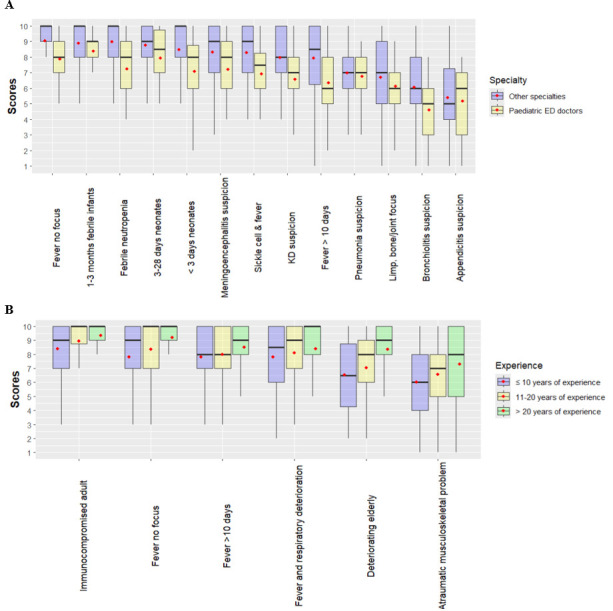
Scores by respondent subgroups: (**A**) For paediatric and neonatal use cases, by respondent specialty, (**B**) for adult use cases, by respondent experience; the dot represents the mean. Alt text: (**A**) Boxplot of paediatric use case scores by specialty (paediatric emergency vs other). Alt text: (**B**) Boxplot of adult use case scores by experience (10 years or less, 11–20 years, over 20 years). ED, emergency department; KD, Kawasaki’s disease.

Figure 3 plots scores across groups. Emergency clinicians score paediatric use cases lower, on average, than other clinicians (panel A); clinicians with less experience score adult use cases lower than those with more experience (panel B).

Both statistical models (online supplemental appendix S3.5) demonstrate a significant relationship between emergency specialty and lower paediatric scores and between having 20+ years of experience and higher adult scores. We find a weak relationship between work setting and adult scores. Other variables were not significant.

### Aetiology preferences

Bacterial aetiology test: 54% of respondents suggested that a ‘generic’ test for bacterial aetiology would be as useful as a test providing results specifying if the bacteria was Gram-positive or Gram-negative. 8% felt that a generic test would not be useful (table 4). There was no evidence of a difference between emergency and other clinicians, consultants and trainees, different experience levels or different work settings (Fisher’s exact test, p=0.134, p=0.182, p=0.424 and p=0.532, respectively) but some evidence (p=0.021) of a difference between Greek and non-Greek respondents, which was not confirmed by an ordinal regression model combining these parameters and adult clinician specialty (which is strongly correlated with practising in Greece).

**Table 4:** Respondents’ preferences regarding testing for more ‘generic’ or specific aetiologies

Response selected for specific questions	Number of respondents	% (95% CI)*
**Question:** ‘For patient use cases for whom a bacterial infection is on the differential, please indicate how useful a test for ‘bacterial’ aetiology would be as compared to a test which provides two results, one for gram positive and one for gram negative bacterial aetiologies.’		
As useful	142	54.0 (48.0 to 60.0)
Useful, but much less useful	101	38.4 (32.6 to 50.0)
Not useful (but a test providing two results would be useful)	17	6.5 (3.8 to 9.7)
None of these tests would be useful	3	1.1 (0.2 to 2.7)
**Any answer**	**263**	**100**
**Question**: ‘For patient use cases for whom an inflammatory aetiology is plausible, and Kawasaki’s Disease (KD) is on the differential, please indicate how useful a test for non-specific inflammatory aetiology (but including KD) would be as compared to a test specific for KD.’		
As useful	37	31.9 (23.7 to 40.5)
Useful, but much less useful	26	22.4 (15.3 to 30.3)
Not useful (but a specific KD test would be useful)	18	15.5 (9.5 to 22.5)
None of these tests would be useful	2	1.7 (0.1 to 4.7)
The most useful option would be a test which showed both results	45	38.8 (30.2, 47.7)
**Any valid answer†**	**116**	**100**

* CI.

† Three respondents were excluded because they said they did not see this type of patient or had contradictory answers (eg, selecting both ‘as useful’ and ‘useful but much less’). 12 respondents ticked more than one answer.

Inflammatory aetiology test: 32% of respondents said that a generic ‘inflammatory’ test would be as useful as a specific KD test, 39% that having both would be the most useful and 15% that a ‘generic’ inflammatory test would not be useful in any situation (alone or combined with a KD test). Sample sizes were too low for subgroup analyses.

### Suggested additional use cases

Free-text suggestions (provided by 14% of respondents) focused primarily on settings, patient profiles, aetiologies and syndromes. Many of the suggestions provided additional nuance to an existing use case. Additional aetiologies/causes of illness suggested for inclusion on a test included: fungal, malignancy, macrophage activation syndrome and endemic zoonoses. Comments highlighted the importance of immune-compromised patients as a target population. Full results are in online supplemental appendix S3.6.

## Discussion

### Principal findings

In a context of quick progress in rapid host-based test development, an improved understanding of user perspectives is essential to bridge between efforts to discover markers for future tests and clinician needs.^5 7^ We therefore developed a Europe-wide online survey of clinicians, successfully reaching 265 clinicians across 30 European countries, with respondents representing a wide range of specialties and subspecialties, experience levels, work settings and professional status. Both paediatric and adult clinicians attributed a relatively high priority to the development of new tests for all listed use cases, giving top scores to children and adults with a fever without a focus, febrile/unwell infants and neonates, and immunocompromised children and adults. These patients have high clinical uncertainty and/or severity risk (although they are not necessarily common presentations) and are therefore often subjected to unnecessary admissions, invasive procedures and second-line antimicrobials.

The relative rank order of the use cases remained stable across all subgroups, indicating a broad consensus on clinical priorities regardless of the respondent’s specific background. Conversely, absolute scores varied slightly by specialty and experience level. In particular, emergency clinicians scored paediatric use cases lower than other clinicians on average, while more experienced clinicians scored adult use cases higher than less experienced clinicians. There was weak evidence that teaching/university hospital clinicians may score adult use cases lower than other clinicians. Emergency paediatricians need to ensure patient flow; hence, they may be less likely to undertake tests than other paediatricians, while adult clinicians in district/regional hospitals may have fewer resources; hence, they may have a higher interest in new tests. In surveying clinicians to understand their potential use of the test, reaching out to the specialties and profiles who are most likely to use it in the future is important in ensuring that the results are relevant.

Although substantial efforts focus on developing tests that can differentiate between Gram-positive and Gram-negative aetiologies, our results suggest that most respondents would find a ‘generic’ bacterial test useful. Similar results were found for a generic inflammatory test. This can help guide discovery efforts and the choice of aetiologies to include in future tests if there are practical limitations in the number that can be included.

Free text comments reinforced the importance of tests for the most vulnerable patients—with immune compromise or complex care needs. Test developers should therefore assess test performance among these subgroups of patients in whom the responses to infections may differ from immunocompetent hosts.^22^

### Strengths and limitations

A major strength of this study was the use of an online survey, which enabled us to reach respondents across a large range of countries across Europe. Our extensive descriptive and statistical analyses of the results (including both use case scores and ranking) and our subgroup analyses using both ordered beta regression and a cumulative link mixed effects model^16 18 23^ have enabled us to extensively check the robustness of our results. This enabled us to address one of the main limitations of the study in relation to our approach to participant selection through professional societies, and the potential bias introduced by the over-representation of certain groups. As clinicians’ choices are likely to be influenced by their specialty and where they work, we performed extensive analysis to look for systematic differences and were reassured to find limited differences in the ranking of use cases and in aetiology preferences across different specialties, and in comparing between Greek and non-Greek respondents. While clinical priorities did appear consistent across our sample, we acknowledge that local variations in infectious disease epidemiology and AMR patterns may influence the eventual real-world utility of a new test in different European regions.

Some studies have also found that respondents to online healthcare personnel surveys are somewhat more likely to be favourable to internet-based decision support than other personnel, though this would likely affect mean scores more than ranking.^24^ Finally, because the survey was disseminated as an ‘open’ survey via professional medical societies, the total number of clinicians reached via these various channels cannot be precisely quantified (due to overlapping memberships and third-party distribution), hence a traditional response rate could not be calculated.

### Implications and future research directions

This study fills a critical research gap concerning Europe’s hospital clinicians’ priorities and preferences. A similar study tailored to community-based physicians, where most patients with fever first present, would be very useful. A large multicentre randomised controlled trial could help better understand the impact of new tests on clinical decision making, which may depend on other tests available and the capacity to act on results. Indeed, Dewez *et al* have found uneven rapid test availability across European hospitals and primary healthcare.^12 25 26^ Finally, it is important to understand whether the new tests should be added to or replace some of the existing tests, or if advocating for increasing the availability of existing tests may sometimes be more cost-effective, particularly in lower-resourced contexts.

## Supplementary material

10.1136/bmjopen-2025-107663 online supplemental file 1

10.1136/bmjopen-2025-107663 online supplemental file 2

10.1136/bmjopen-2025-107663 online supplemental file 3

## Data Availability

Data are available in a public, open access repository.
