# Supplementary material for: Priorities for the development of a new rapid diagnostic test for patients with fever: a cross-sectional online survey among hospital physicians across Europe
Source: BMJ Open. 2026 Mar 24;16(3):e107663. doi: 10.1136/bmjopen-2025-107663 (PMC13034235; doi:10.1136/bmjopen-2025-107663)
Supplement: online supplemental file 1 [file bmjopen-16-3-s001.docx]

**In which patients presenting with fever do we most need a new diagnostic test? An online survey of hospital doctors across Europe**

**Authors**: Gabrielle Bonnet, Maryke Nielsen, Anna M. Foss, Alexandra Lewin, Ruud G. Nijman, Elizabeth Fitchett, Enitan D. Carrol, Shunmay Yeung, and the DIAMONDS consortium

**Corresponding authors:**

Gabrielle Bonnet, PhD, ORCID: 0000-0002-3539-5001

Affiliation: London School of Hygiene & Tropical Medicine

Address: Keppel Street, London, WC1E 7HT, United Kingdom

Email: [gabrielle.bonnet@lshtm.ac.uk](mailto:gabrielle.bonnet@lshtm.ac.uk)

Shunmay Yeung, PhD, ORCID: 0000-0002-0997-0850

Affiliations: London School of Hygiene & Tropical Medicine; St. Mary’s Imperial College Hospital, London

Addresses: Keppel Street, London, WC1E 7HT, United Kingdom; St Mary's Hospital, Praed St, London W2 1NY

Email: [shunmay.yeung@lshtm.ac.uk](mailto:shunmay.yeung@lshtm.ac.uk)

**Contents**

[In which patients presenting with fever do we most need a new diagnostic test? An online survey of hospital doctors – Appendix 1](#_Toc200208478)

[S1. Survey tools 1](#_Toc200208479)

[S2. Sample size calculations 5](#_Toc200208480)

[S3. Supplementary analyses, tables and graphs 6](#_Toc200208481)

[S3.1. Comparison of respondent numbers with target population 6](#_Toc200208482)

[S3.2 Additional descriptive results 8](#_Toc200208483)

[S3.3 Ranking significance 9](#_Toc200208484)

[S3.4 Differences in ranking by subgroup 10](#_Toc200208485)

[S3.5 Differences in overall scoring levels by subgroup 11](#_Toc200208486)

[S3.6. Qualitative feedback on priorities for test development 16](#_Toc200208487)

[S4. CROSS checklist 17](#_Toc200208488)

## **S1. Survey tools**

Below is the survey tool that was made available on the Online Surveys (JISC) version 3 platform. No technical characteristic prevented respondents from giving multiple responses, but it was expected that there was limited incentive to re-take the survey (barring technical issues preventing submission the first time).

**Identifying a multi-aetiologies test with the best value for money for patients with fever and uncertain diagnosis**

**Information and consent**

You are invited to take part in a survey to inform the development and assessment of new host biomarker-based tests for patient management in hospitals. This survey, led by an EU-funded consortium, DIAMONDS (https://www.diamonds2020.eu) will help us prioritize which patient types and features test development should focus on. it is undertaken by a team from the London School for Hygiene and Tropical Medicine.

The survey should take approximately 10 minutes. Answers are automatically saved for you to come back later if needed.

There is no obligation to participate. We are not assessing the quality of your work or institution. Your responses will only be reported in aggregate, and the fully anonymized database may be shared via a public data repository or directly with other researchers.

We do not anticipate any risks associated with your participation, but feel free to contact us with any questions or concerns.

**Please answer below to confirm that you consent to your data being used in the ways described above and are working in relevant settings**

1. I voluntarily agree to participate in this research study and consent to my data being used as described above:

□ Yes

□ No

2. I confirm that I am a doctor working in a hospital in Europe (including non-EU countries) and am involved in patient assessment and diagnosis:

□ Yes

□ No

**QUESTIONNAIRE**

**Part 1: General information**

1. Which age-group of patients do you work with on a routine basis (tick all those that apply)? *[note: will serve as a filter to show only relevant questions in the following parts]*

□ Neonates

□ Children

□ Adults

1. What is your specialty? (please tick one main specialty/sub-specialty)

□ Paediatrics/neonatology

□ General or no specialty

□ Emergency

□ Intensive care

□ Neonatology

□ Infectious Disease/Immunology

□ Respiratory medicine

□ Gastroenterology

□ Rheumatology

□ Haematology/Oncology

□ Surgery

□ Other (please specify) ________________

□ Adults

□ General/Ambulatory

□ Emergency

□ Intensive care

□ Obstetrics/Gynaecology

□ Infectious Disease/Immunology

□ Respiratory medicine

□ Gastroenterology

□ Rheumatology

□ Haematology/Oncology

□ Surgery

□ Other (please specify) ________________

1. How many years have you been working as a clinician?

□ 1-2 years

□ 3-4 years

□ 5-10 years

□ 11-20 years

□ > 20 years

1. Are you:

□ An intern/trainee

□ Consultant/attending physician

□ Medical Officer

□ Clinical Officer

□ Other

1. What type of institution do you work in?

□ Regional/District hospital

□ Teaching/University hospital

□ Other health facility/comments (please specify if needed) _________

1. In which country is your institution located? *[drop-down menu of countries]*

The country designations employed above do not imply the expression of any opinion whatsoever concerning the legal designation or status of any country.

**Part 2: Prioritizing target patients for a new multi-aetiologies test**

For each use case below, please indicate how important you think it is as a target for the development of a new multi-aetiologies test on a scale from 1 to 10 (10 corresponding to very high importance). Aetiologies may include: viral, bacterial (non-specific and/or gram positive/gram negative), tuberculosis, inflammatory disease (non-specific) and Kawasaki’s Disease. *[Note: each use case was associated with a drop-down menu with 11 options: a rating from 1 to 10, and the option to answer “I do not see this type of patient”]*

1. **Neonates**

1a. “Unwell neonate – early onset” (< 3 days) - Differential includes infection, ischemic-hypoxic encephalopathy, congenital malformation etc.* *[drop-down menu]*

*Example of presentation: irritable, lethargic, not feeding well, apnoea, abnormal heart or respiratory rate/pattern, temperature dysregulation, altered muscle tone, vomiting, respiratory distress, seizures, need for cardiopulmonary support.

1b. “Unwell neonate – later onset” (3-28 days) - Differential includes sepsis, congenital malformation etc.* *[drop-down menu]*

**Example of presentation: as for early onset plus persistent pulmonary hypertension, unexplained excessive bleeding, thrombocytopenia, abnormal coagulation, altered glucose homeostasis, metabolic acidosis.*

1. **Paediatric patients (children without any specific co-morbidity)**

2a. Very young febrile infant (1-3 months) *[drop-down menu]*

2b. Bronchiolitis (<18 months) with or without bacterial infection *[drop-down menu]*

2c. Child for whom Kawasaki’s Disease is on the differential *[drop-down menu]*

2d. Unwell or deteriorating child with a fever without focus* *[drop-down menu]*

** Example of presentation: children presenting with fever and “red flag” symptom/signs who do not have an obvious respiratory focus or features of Kawasaki’s disease*

2e. Prolonged fever (> 10 days) – unclear aetiology *[drop-down menu]*

2f. Child with suspicion of meningoencephalitis *[drop-down menu]*

2g. Suspected pneumonia *[drop-down menu]*

2h. Possible appendicitis *[drop-down menu]*

2i. Atraumatic limp or bone/joint focus with or without a fever* *[drop-down menu]*

**Example of presentation: acute joint swelling and raised inflammatory markers with or without fever without a known traumatic source.*

1. **Paediatric patients with co-morbidities**

3a. Febrile neutropenic child (and other immunocompromised children) *[drop-down menu]*

3b. Child with sickle cell disease and fever *[drop-down menu]*

1. **Adult patients**

4a. Fever without a focus, possible sepsis *[drop-down menu]*

4b. Prolonged fever (>10 days) – unclear aetiology *[drop-down menu]*

4c. Fever and respiratory deterioration – possible infection, embolism or cardiac aetiology *[drop-down menu]*

4d. Immunocompromised adult with fever *[drop-down menu]*

4e. Atraumatic musculoskeletal problem with or without a fever* *[drop-down menu]*

**Example of presentation: acute joint swelling and raised inflammatory markers with or without fever without a known traumatic source.*

4f. Elderly patient with a sudden non-specific deterioration e.g. falls, confusion, lethargy *[drop-down menu]*

1. **Any high priority use cases we have missed or overall comments?** _________________________________

**Part 3: relative importance of including more vs. less specific aetiologies in the test**

We may not be able to add all aetiologies on the test and may need to prioritize. In this context, please answer the following:

1. For patient use cases for whom a bacterial infection is on the differential, please indicate how useful a test for ‘bacterial’ aetiology would be as compared to a test which provides two results, one for gram positive and one for gram negative bacterial aetiologies**:** *[note: only one option could be ticked]*

□ As useful

□ Useful, but much less useful

□ Not useful (but a test providing two results would be useful)

□ None of these tests would be useful

2. For patient use cases for whom an inflammatory aetiology is plausible, and Kawasaki's disease (KD) is on the differential, please indicate how useful a test for non-specific inflammatory aetiology (but including KD) would be as compared to a test specific for KD (tick all that apply):

□ As useful

□ Useful, but much less useful

□ Not useful (but a specific KD test would be useful)

□ The most useful option would be a test which showed both results

□ None of these tests would be useful

□ This type of test is not relevant to the patients I see

**Optional question**

Are you aware of the DIAMONDS project?

□ Yes

□ No

If yes, how have you been informed about this project? (you can tick more than one answer)

□ Presentations of DIAMONDS’ research during our Society’s annual meeting

□ Presentations during local/national pediatric infectious diseases meeting(s) held in my country

□ Recently published articles in the literature

□ Messages in social media

□ Visited the consortium’s website

□ Other (please specify)

If you replied "Other", could you please specify? _________________________________________

**Thank you very much for your time! This is the end of the survey.**

## **S2. Sample size calculations**

Sample sizes were estimated based on the results of the pilot, which included 16 clinicians: 11 infectious disease specialists, four generalists/doctors with no specialty, and one rheumatologist. six of these respondents had up to 10 years of experience and seven 20 years or more. Two respondents worked in regional/district hospitals. Pilot respondents mostly worked with children (14) and or neonates (9), two worked with adults. The number of adult clinicians in the pilot was small so calculations were mostly based on paediatric respondents.

**Sample size needed for the scoring of use cases**. The average scores of the paediatric/neonatal use cases in the pilot ranged between 6.1 and 9.5 (between 4 and 10 if adult use cases were included), with an average difference between successive use cases of 0.3 points overall, but only 0.2 between higher ranked use cases (top half). The standard deviation in answers ranged between 0.9 and 2.3 points across all paediatric and neonatal use cases, with a lower standard deviation (1.2 points on average) for the top ranked use cases. We have more interest in understanding ranking among the highest ranked than among the lowest ranked use cases, as the former may translate into priorities for test development. In particular, we intend to select the top 3 use cases for our work within the DIAMONDS consortium. In this context, we made the following assumptions:

- The top priority has an average score around 9.5.
- The standard deviation is 1.2 points.
- We want to be able to identify a difference of 0.6 points or more in the average scores of two use cases (i.e., the difference between the first and fourth ranked use case) with 80% power and a confidence level of at least 90%.

We used the R BetaPASS library to calculate the sample size needed under those conditions. The minimum sample size is 69 respondents. Meanwhile, 87 respondents would allow us to identify the same effect (0.6 points difference in scores) with 80% power and a confidence level of 0.05.

While the minimum sample size is 69 respondents replying to each use case, most clinicians do not work across all age groups. Assuming the adult clinician sample is different from the paediatric sample, and that around 64% of paediatricians also work with neonates (in line with figures from the pilot), we would need a minimum sample of 177 respondents in total across all specialties and age groups. If in addition we assume around 5% of missing answers, then we will need a large sample size (73 per use case – 92 for a confidence level of 0.05 – and 186 respondents in total).

A larger sample size is also desirable for subgroup analyses. To assess ranking within a subgroup, for example, with the same requirements as described above, we would need 69 respondents in that subgroup. A minimum size of 22 respondents is needed to identify a difference in scores of 1.2 points with 80% power and a confidence level of 0.1. Such a difference in scores is approximately 6 ranks, or the difference between the top ranked use case and a medium-ranked use case, which would mean very large differences in scores across subgroups, hence we will not compare ranks across subgroups when one of those has fewer than 22 patients.

**Questions on aetiology**. Those are multiple choice questions regarding clinicians’ preference between more or less specific tests. There are 4 mutually exclusive options for the first aetiology question, and 5 options (with the possibility to select more than one answer) for the second question. We are most interested in 1) the proportion of respondents who believe that a “non-specific” test (for bacterial or inflammatory aetiology) has at least some utility (alone or combined with another test), 2) the proportion of respondents who believe it is as useful as more specific tests. To be able to measure these proportions with a margin of error of 10% and a confidence level of 90%, we would need a minimum sample size of 69 respondents, and 97 to estimate the proportions with a margin of error of 10% and a confidence level of 95%. Most subgroup analyses are unlikely to be sufficiently powered: a sample size of at least 75 respondents in each subgroup would be needed to identify a difference of 20 percentage points with 80% power and 90% confidence, so we may not be able to undertake much subgroup analysis.

## **S3. Supplementary analyses, tables and graphs**

### **S3.1. Comparison of respondent numbers with target population**

S3.1 Table compares the numbers of hospital-based paediatricians and adult clinicians with sample sizes achieved. The ‘expected’ samples correspond to the number of respondents expected from each country given the achieved sample size, if numbers had been fully representative of clinicians in each country. We excluded Russia and Ukraine from calculations, so that our expected sample size is only representative of Europe minus these two countries. The paediatric sample is not too dissimilar from the expected sample, despite some over-representation of countries such as Greece and Switzerland. On the other hand, the adult sample is highly skewed, with almost three quarters of the sample coming from Greece.

**S3.1 Table: comparison between country clinician numbers and numbers in sample**

| **Country** | **Paediatricians in sample** | **Adult clinicians in sample** | **Expected paediatric sample size (excl. Ukraine, Russia)** | **Expected adult sample size (excl. Ukraine, Russia)** | **Estimated number of hospital-based clinicians (all ages)** | **Derived from*** | **Estimated number of hospital-based paediatricians** | **Derived from**** |
| --- | --- | --- | --- | --- | --- | --- | --- | --- |
| Greece | 19 | 105 | 3.3 | 2.1 | 24408 | Statista | 2130 | Dewez et al. |
| Netherlands | 12 | 15 | 2.7 | 1.6 | 23982 | Statista | 1751 | Dewez et al. |
| United Kingdom | 16 | 9 | 16.1 | 10.9 | 158282 | Statista | 10464 | Dewez et al. |
| Italy | 14 | 1 | 17.5 | 9.4 | 138530 | Statista | 11354 | Dewez et al. |
| Spain | 12 | 0 | 11.7 | 8.9 | 121799 | Statista | 7589 | Dewez et al. |
| Switzerland | 10 | 0 | 1.3 | 2.2 | 30289 | Statista | 839 | Dewez et al. |
| Turkey | 8 | 0 | 12.3 | 10.3 | 148154 | Statista | 8022 | Hospital clinician number |
| Germany | 6 | 0 | 12.2 | 15.0 | 211941 | Statista | 7924 | Dewez et al. |
| Poland | 6 | 0 | 15.2 | 5.1 | 79642 | Eurostats | 9905 | Dewez et al. |
| Slovenia | 0 | 6 | 0.6 | 0.3 | 4121 | Statista | 396 | Dewez et al. |
| Sweden | 6 | 0 | 1.7 | 1.9 | 27419 | Eurostats | 1083 | Dewez et al. |
| France | 4 | 0 | 10.2 | 12.9 | 182556 | Statista | 6622 | Dewez et al. |
| Belgium | 2 | 1 | 1.2 | 1.6 | 23072 | Statista | 781 | Dewez et al. |
| Cyprus | 1 | 2 | 0.1 | 0.2 | 2709 | Eurostats | 68 | Dewez et al. |
| Hungary | 3 | 0 | 2.2 | 1.0 | 15463 | Statista | 1432 | Dewez et al. |
| Portugal | 3 | 0 | 3.2 | 1.8 | 26072 | Statista | 2085 | Dewez et al. |
| Austria | 2 | 0 | 1.2 | 1.9 | 26046 | Statista | 774 | Dewez et al. |
| Georgia | 2 | 0 | 0.7 | 0.6 | 8715 | Country population | 472 | Hospital clinician number |
| Ireland | 2 | 0 | 0.7 | 0.7 | 10188 | Statista | 451 | Dewez et al. |
| North Macedonia | 2 | 0 | 0.3 | 0.3 | 3873 | Eurostats | 210 | Hospital clinician number |
| Norway | 2 | 0 | 1.3 | 1.1 | 15187 | Statista | 875 | Dewez et al. |
| Romania | 2 | 0 | 4.1 | 2.8 | 41139 | Eurostats | 2655 | Dewez et al. |
| Albania | 1 | 0 | 0.6 | 0.5 | 6607 | Country population | 358 | Hospital clinician number |
| Armenia | 1 | 0 | 0.5 | 0.5 | 6559 | Country population | 355 | Hospital clinician number |
| Bulgaria | 0 | 1 | 2.3 | 1.2 | 18114 | Eurostats | 1475 | Dewez et al. |
| Croatia | 0 | 1 | 0.9 | 0.6 | 8986 | Eurostats | 583 | Dewez et al. |
| Denmark | 1 | 0 | 0.7 | 1.4 | 20017 | Statista | 469 | Dewez et al. |
| Estonia | 1 | 0 | 0.3 | 0.2 | 3064 | Statista | 166 | Hospital clinician number |
| Finland | 1 | 0 | 1.0 | 0.7 | 10769 | Statista | 623 | Dewez et al. |
| Latvia | 1 | 0 | 0.4 | 0.2 | 3511 | Statista | 238 | Dewez et al. |
| Andorra | 0 | 0 | 0.0 | 0.0 | 186 | Country population | 10 | Hospital clinician number |
| Azerbaijan | 0 | 0 | 2.0 | 1.7 | 23824 | Country population | 1290 | Hospital clinician number |
| Belarus | 0 | 0 | 1.8 | 1.5 | 21861 | Country population | 1184 | Hospital clinician number |
| Bosnia and Herzegovina | 0 | 0 | 0.6 | 0.5 | 7687 | Country population | 416 | Hospital clinician number |
| Czechia | 0 | 0 | 1.0 | 2.0 | 27800 | Statista | 669 | Dewez et al. |
| Iceland | 0 | 0 | 0.1 | 0.1 | 1012 | Statista | 55 | Hospital clinician number |
| Kazakhstan | 0 | 0 | 3.8 | 3.1 | 45100 | Country population | 2442 | Hospital clinician number |
| Kosovo | 0 | 0 | 0.3 | 0.3 | 4197 | Country population | 227 | Hospital clinician number |
| Liechtenstein | 0 | 0 | 0.0 | 0.0 | 82 | Eurostats | 4 | Hospital clinician number |
| Lithuania | 0 | 0 | 1.0 | 0.6 | 8402 | Statista | 676 | Dewez et al. |
| Luxembourg | 0 | 0 | 0.1 | 0.1 | 1504 | Country population | 81 | Hospital clinician number |
| Malta | 0 | 0 | 0.1 | 0.1 | 1381 | Eurostats | 81 | Dewez et al. |
| Moldova | 0 | 0 | 0.5 | 0.4 | 6100 | Country population | 330 | Hospital clinician number |
| Monaco | 0 | 0 | 0.0 | 0.0 | 86 | Country population | 5 | Hospital clinician number |
| Montenegro | 0 | 0 | 0.1 | 0.1 | 1066 | Eurostats | 58 | Hospital clinician number |
| Russia | 0 | 0 | NA | NA | 340155 | Country population | 18418 | Hospital clinician number |
| San Marino | 0 | 0 | 0.0 | 0.0 | 79 | Country population | 4 | Hospital clinician number |
| Serbia | 0 | 0 | 1.1 | 0.9 | 12694 | Eurostats | 687 | Hospital clinician number |
| Slovakia | 0 | 0 | 1.0 | 0.9 | 12292 | Eurostats | 666 | Hospital clinician number |
| Ukraine | 0 | 0 | NA | NA | 115169 | Paediatrician number | 6236 | Dewez et al. |
| Vatican City | 0 | 0 | 0.0 | 0.0 | 49 | Country population | 3 | Hospital clinician number |

* The total number of hospital-based clinicians was deduced, if possible, from Statista figures on hospital employment and the share of physicians within it. Otherwise, figures were derived from Eurostat data on the number of doctors per country, using a proportion based on countries with data, when even this approach was not possible but paediatric doctor information was available, a proportionality helped obtain total hospital-based physician information. Finally, when none of the above worked, the number of hospital-based physicians was estimated based on the country’s total population. ** The number of hospital-based paediatricians was taken, if possible, from Dewez et al., 2022 ^1^. otherwise, it was derived from the total number of hospital clinicians, using a proportion based on countries with data.

Table S3.2 compares the proportions of clinicians by experience, status, work setting and specialty in our samples with figures from different countries in Europe. We often did not find comprehensive information, making it harder to know when a subgroup is over-/under-represented. However, infectious disease clinicians appear largely over-represented in the sample, while emergency clinicians are under-represented in the adult sample. Larger hospitals and consultants may also be somewhat over-represented.

**S3.2 Table: comparison between expected numbers in other subgroups and numbers in sample**

| **Subgroup** | **Proportions in different countries of Europe** | **Achieved (paediatricians)** | **Achieved (adult doctors)** |
| --- | --- | --- | --- |
| Less than 10 years of experience | 20%: Dewez et al ^1^, Eurostat ^2^ assuming clinicians start practising around 25 years old | 32.5% | 22.4% |
| Less than 20 years of experience | 40-50% (Eurostat ^2^) | 62% | 64% |
| Consultants/attending clinicians | France: 73% ^3^, Belgium: 60% ^4^, UK: 41% ^5^ | 79% | 82% |
| Share of visits or staff in university or teaching hospitals | France: 35% of activity ^6^, UK: 40-50% of visits ^7^, Switzerland: 24% of staff ^7^ | 82% | 56% |
| Share of emergency clinicians | France: 17.4% ^8^, UK: 12% of non-surgical/anaesthesiologist/psychiatric doctors ^5^ | 20% | 1% |
| Share of infectious disease clinicians | UK: 5.4% ^5^, Europe: variable from around 0-6% ^9^ | 46% | 60% |

### **S3.2 Additional descriptive results**

#### S3.2.1. Non-response among clinicians seeing a specific age range

S3.3. Table shows the proportions of clinicians not rating a specific use case despite routinely seeing the broader age group (neonates, children or adults). All these percentages are below 5% (most often substantially below), but one use case stands out: the youngest unwell neonates. Indeed, among our respondents regarding the neonatal age group, a substantial proportion were not neonatologists but paediatricians, who would often not see the youngest neonates who have never left the neonatal units.

**S.3.3. Table: proportion of clinicians not rating the use case despite routinely seeing the corresponding age group**

| Use case | Proportion not rating the use case among clinicians routinely seeing the corresponding age group |
| --- | --- |
| 1a. “Unwell neonate – early onset” (<3 days) | 8.8% |
| 1b. “Unwell neonate – later onset” (3-28 days) | 2.2% |
| 2a. Very young febrile infant (1-3 months) | 0.8% |
| 2b. Bronchiolitis (<18 months) with or without bacterial infection | 0.8% |
| 2c. Child for whom Kawasaki’s Disease is on the differential | 1.7% |
| 2d. Unwell or deteriorating child with a fever without focus | 0.8% |
| 2e. Prolonged fever (>10 days) – unclear aetiology | 0.8% |
| 2f. Child with suspicion of meningoencephalitis | 2.5% |
| 2g. Suspected pneumonia | 0.8% |
| 2h. Possible appendicitis | 3.4% |
| 2i. Atraumatic limp or bone/joint focus with or without a fever | 2.5% |
| 3a. Febrile neutropenic child (and other immunocompromised children) | 1.7% |
| 3b. Child with sickle cell disease and fever | 5.0% |
| 4a. Fever without a focus, possible sepsis | 0.7% |
| 4b. Prolonged fever (>10 days) – unclear aetiology | 0.7% |
| 4c. Fever and respiratory deterioration – possible infection, embolism or cardiac aetiology | 1.4% |
| 4d. Immunocompromised adult with fever | 2.1% |
| 4e. Atraumatic musculoskeletal problem with or without a fever | 4.3% |
| 4f. Elderly patient with a sudden non-specific deterioration e.g. falls, confusion, lethargy | 3.6% |

#### S3.2.2. Differences in scores

To understand differences in scores, we compared how clinicians scored each of the paediatric and neonatal use cases compared with the highest scored paediatric use case (fever without a focus with amber/red flag signs). S3.4 table provides, for each use case, the number of respondents that 1) scored it lower than the “fever without a focus” use case, 2) scored it the same, and 3) scored it higher. For example, when comparing the fever without a focus and 1-3 months old febrile infant use cases, 61 of 120 respondents gave both use cases the same score. Out of those that gave different scores to the two use cases, almost the same number placed one or the other use case on top (30 vs. 29). However, fewer than 5% of respondents scored the bottom two use cases (bronchiolitis and appendicitis suspicion) higher than “fever without a focus” and 80% gave fever without a focus a strictly higher score.

**S3.4 Table: Relative rating of different paediatric use cases as compared to the highest rated paediatric use case (fever without a focus)**

| **Use case** | **Number of respondents giving the use case a lower score** | **Number of respondents giving the use case the same score** | **Number of respondents giving the use case a higher score** | **Total number of respondents*** |
| --- | --- | --- | --- | --- |
| 1-3 months febrile infant | 30 | 61 | 29 | 120 |
| Febrile neutropenia | 38 | 55 | 27 | 120 |
| 3-28 days neonates | 23 | 41 | 17 | 81 |
| <3 days neonates | 23 | 34 | 24 | 81 |
| Meningoencephalitis suspicion | 44 | 53 | 21 | 118 |
| Sickle cell & fever | 56 | 42 | 21 | 119 |
| KD suspicion | 59 | 47 | 14 | 120 |
| Fever > 10 days | 58 | 46 | 16 | 120 |
| Pneumonia suspicion | 85 | 23 | 12 | 120 |
| Limp, bone/joint focus | 83 | 28 | 8 | 119 |
| Bronchiolitis suspicion | 99 | 17 | 4 | 120 |
| Appendicitis suspicion | 94 | 19 | 5 | 118 |

* There were virtually no missing answers to use case questions but in 4.4% and 1.9% of answers to neonatal and paediatric use cases respectively, respondents stated they did not manage that specific use case, hence totals differ from the total number of respondents that work with children/neonates.

Similarly, for adult respondents, S3.5 Table compares responses to the “immunocompromised adult” use case (highest scored, on average, out of all use cases) and other adult use cases. Results are less extreme than in the case of paediatric use cases. However, when considering, for example, the lowest scored adult use case (atraumatic musculoskeletal problems), we see that this use case is scored lower than immunocompromised adults by 59% of respondent, and more highly scored by only 12% of them.

**S3.5 Table: Relative scores of different adult use cases as compared to the highest scored adult use case (immunocompromised adult)**

| **Use case** | **Number of respondents giving the use case a lower score** | **Number of respondents giving the use case the same score** | **Number of respondents giving the use case a higher score** | **Total number of respondents*** |
| --- | --- | --- | --- | --- |
| Fever without a focus | 40 | 77 | 21 | 138 |
| Fever > 10 days | 65 | 56 | 17 | 138 |
| Fever & respiratory deterioration | 59 | 60 | 18 | 137 |
| Deteriorating elderly | 81 | 40 | 16 | 137 |
| Atraumatic musculoskeletal problem | 92 | 33 | 11 | 136 |

* Note that, in 1.1% of answers to adult use cases, respondents stated they did not manage that specific use case, so total numbers of respondents may differ by 1-2 individuals.

### **S3.3 Ranking significance**

Tables S3.6 to S3.8 provide the adjusted p-values for all pairwise comparisons between use cases belonging to different age groups: paediatric, paediatric and neonatal, and adult only. All p-values for the difference in ranks was computed using only cases with complete answers and Eisinga, Heskes, Pelzer & Te Grotenhuis’s ^10^ all-pairs test, adjusted using the Benjamini-Hochberg method ^11^. p-values above 0.1 are highlighted in dark pink/orange and p-values between 0.05 and 0.1 in light pink.

**Table S3.6. Adjusted p-values for all pairwise comparisons between paediatric use cases, using complete paediatric answers only (N=106)**

| **Use case** | **Q2d** | **Q2a** | **Q3a** | **Q2f** | **Q3b** | **Q2c** | **Q2e** | **Q2g** | **Q2i** | **Q2h** | **Q2b** |
| --- | --- | --- | --- | --- | --- | --- | --- | --- | --- | --- | --- |
| **Q2d: Fever no focus** |  | 0.99175 | 0.43353 | 0.01542 | 0.00079 | 1.10E-05 | 1.70E-05 | 1.90E-12 | <2e-16 | <2e-16 | <2e-16 |
| **Q2a: 1-3 months febrile infant** | 0.99175 |  | 0.43759 | 0.01598 | 0.00083 | 1.20E-05 | 1.80E-05 | 2.10E-12 | <2e-16 | <2e-16 | <2e-16 |
| **Q3a: Febrile neutropenia** | 0.43353 | 0.43759 |  | 0.11423 | 0.01229 | 0.00036 | 0.00051 | 6.30E-10 | 1.60E-13 | <2e-16 | <2e-16 |
| **Q2f: Meningoencephalitis suspicion** | 0.01542 | 0.01598 | 0.11423 |  | 0.38105 | 0.05426 | 0.06768 | 8.50E-06 | 1.30E-08 | <2e-16 | <2e-16 |
| **Q3b: Sickle cell & fever** | 0.00079 | 0.00083 | 0.01229 | 0.38105 |  | 0.32828 | 0.37704 | 0.00041 | 2.80E-06 | 2.4E-13 | 5.20E-14 |
| **Q2c: KD suspicion** | 1.10E-05 | 1.20E-05 | 0.00036 | 0.05426 | 0.32828 |  | 0.94297 | 0.01354 | 0.0003 | 5E-10 | 1.3E-10 |
| **Q2e: Fever > 10 days** | 1.70E-05 | 1.80E-05 | 0.00051 | 0.06768 | 0.37704 | 0.94297 |  | 0.01048 | 0.0002 | 2.6E-10 | 6.8E-11 |
| **Q2g: Pneumonia suspicion** | 1.90E-12 | 2.10E-12 | 6.30E-10 | 8.50E-06 | 0.00041 | 0.01354 | 0.01048 |  | 0.28348 | 0.00034 | 0.00015 |
| **Q2i: Limp, bone/joint focus** | <2e-16 | <2e-16 | 1.60E-13 | 1.30E-08 | 2.80E-06 | 0.0003 | 0.0002 | 0.28348 |  | 0.01489 | 0.00836 |
| **Q2h: Appendicitis suspicion** | <2e-16 | <2e-16 | <2e-16 | <2e-16 | 2.40E-13 | 5.00E-10 | 2.60E-10 | 0.00034 | 0.01489 |  | 0.85928 |
| **Q2b: Bronchiolitis suspicion** | <2e-16 | <2e-16 | <2e-16 | <2e-16 | 5.20E-14 | 1.30E-10 | 6.80E-11 | 0.00015 | 0.00836 | 0.85928 |  |

**Table S3.7. Adjusted p-values for all pairwise comparisons between paediatric and neonatal use cases, using respondents with complete paediatric and neonatal answers only (N=69)**

| **Use case** | **Q2d** | **Q3a** | **Q2a** | **Q1b** | **Q1a** | **Q2f** | **Q3b** | **Q2c** | **Q2e** | **Q2g** | **Q2i** | **Q2h** | **Q2b** |
| --- | --- | --- | --- | --- | --- | --- | --- | --- | --- | --- | --- | --- | --- |
| **Q2d: Fever no focus** |  | 0.65861 | 0.61093 | 0.35405 | 0.12711 | 0.03281 | 0.00969 | 0.00036 | 0.00015 | 6.3E-08 | 3.5E-09 | <2e-16 | <2e-16 |
| **Q3a: Febrile neutropenia** | 0.65861 |  | 0.9391 | 0.65861 | 0.31141 | 0.10299 | 0.03746 | 0.00225 | 0.00113 | 1.1E-06 | 7.7E-08 | 3.8E-15 | 2.8E-16 |
| **Q2a: 1-3 months febrile infant** | 0.61093 | 0.9391 |  | 0.69716 | 0.34741 | 0.12005 | 0.04602 | 0.00291 | 0.00152 | 1.6E-06 | 1.2E-07 | 7E-15 | 4.9E-16 |
| **Q1b: Neonates 3-28 days** | 0.35405 | 0.65861 | 0.69716 |  | 0.60344 | 0.24765 | 0.11664 | 0.01083 | 0.00595 | 0.000012 | 1.4E-06 | 2.4E-13 | 1.6E-14 |
| **Q1a: Neonates 1-3 days** | 0.12711 | 0.31141 | 0.34741 | 0.60344 |  | 0.57983 | 0.32914 | 0.0532 | 0.03281 | 0.00019 | 0.000026 | 3.1E-11 | 2.7E-12 |
| **Q2f: Meningoencephalitis suspicion** | 0.03281 | 0.10299 | 0.12005 | 0.24765 | 0.57983 |  | 0.68989 | 0.1901 | 0.12711 | 0.00225 | 0.00041 | 2.9E-09 | 3.2E-10 |
| **Q3b: Sickle cell & fever** | 0.00969 | 0.03746 | 0.04602 | 0.11664 | 0.32914 | 0.68989 |  | 0.37302 | 0.28358 | 0.00865 | 0.0022 | 4.5E-08 | 5.9E-09 |
| **Q2c: KD suspicion** | 0.00036 | 0.00225 | 0.00291 | 0.01083 | 0.0532 | 0.1901 | 0.37302 |  | 0.84658 | 0.09997 | 0.0361 | 0.000008 | 1.6E-06 |
| **Q2e: Fever > 10 days** | 0.00015 | 0.00113 | 0.00152 | 0.00595 | 0.03281 | 0.12711 | 0.28358 | 0.84658 |  | 0.14306 | 0.05811 | 0.000022 | 4.8E-06 |
| **Q2g: Pneumonia suspicion** | 0.00000006 | 1.1E-06 | 1.6E-06 | 0.000012 | 0.00019 | 0.00225 | 0.00865 | 0.09997 | 0.14306 |  | 0.68258 | 0.00827 | 0.00291 |
| **Q2i: Limp, bone/joint focus** | 3.5E-09 | 7.7E-08 | 1.2E-07 | 1.4E-06 | 0.000026 | 0.00041 | 0.0022 | 0.0361 | 0.05811 | 0.68258 |  | 0.03044 | 0.0121 |
| **Q2h: Appendicitis suspicion** | <2e-16 | 3.8E-15 | 7E-15 | 2.4E-13 | 3.1E-11 | 2.9E-09 | 4.5E-08 | 0.000008 | 0.000022 | 0.00827 | 0.03044 |  | 0.77139 |
| **Q2b: Bronchiolitis suspicion** | <2e-16 | 2.8E-16 | 4.9E-16 | 1.6E-14 | 2.7E-12 | 3.2E-10 | 5.9E-09 | 1.6E-06 | 0.0000048 | 0.00291 | 0.0121 | 0.77139 |  |

**Table S3.8. Adjusted p-values for all pairwise comparisons between adult use cases, using respondents with complete adult answers only (N=129)**

| **Use case** | **Q4d** | **Q4a** | **Q4c** | **Q4b** | **Q4f** | **Q4e** |
| --- | --- | --- | --- | --- | --- | --- |
| **Q4d: Immuno-compromised adult** |  | 0.13052 | 0.0027 | 0.00018 | 6.50E-11 | <2e-16 |
| **Q4a: Fever no focus** | 0.13052 |  | 0.12296 | 0.02351 | 6.60E-07 | 4.60E-13 |
| **Q4c: Fever & respiratory deterioration** | 0.0027 | 0.12296 |  | 0.45435 | 0.0008 | 2.00E-08 |
| **Q4b: Fever > 10 days** | 0.00018 | 0.02351 | 0.45435 |  | 0.00895 | 1.10E-06 |
| **Q4f: Deteriorating elderly** | 6.50E-11 | 6.60E-07 | 0.0008 | 0.00895 |  | 0.03073 |
| **Q4e: Atraumatic musculoskeletal problem** | <2e-16 | 4.60E-13 | 2.00E-08 | 1.10E-06 | 0.03073 |  |

### **S3.4 Differences in ranking by subgroup**

Tables S3.9 and S3.10 show the ranking of use cases for each subgroup. They are highly similar across subgroups. Whenever two use cases were ranked differently in two subgroups, the differences in rank were never significant in both subgroups (i.e. we never found that use case 1 was ranked significantly higher than use case 2 in subgroup A but significantly lower in subgroup B). The sole purpose of the colour code is to help the reader read the table more easily (seeing rapidly how the location of use cases differs across subgroups).

**Table S3.9: Differences in rankings of paediatric use cases across respondent subgroups (CLD notation*)**

| **Emergency (ED) clinicians** | | **Non-ED clinicians** | | | **1-10 years of experience** | | **11-20 years of experience** | | **Over 20 years of experience** | | **Teaching or university hospital** | | **District or regional hospital** | |
| --- | --- | --- | --- | --- | --- | --- | --- | --- | --- | --- | --- | --- | --- | --- |
| **Use case** | **CLD** | **Use case** | **CLD** | **Use case** | | **CLD** | **Use case** | **CLD** | **Use case** | **CLD** | **Use case** | **CLD** | **Use case** | **CLD** |
| 2a. 1-3 months febrile infant | a | 2d | a | 2d | | a | 2a | a | 2a | a | 2d | a | 2d | a |
| 2d. Fever no focus, child | ab | 3a | ab | 3a | | a | 2d | a | 2d | a | 2a | a | 2a | a |
| 3a. Febrile neutropenia | abc | 2a | ab | 2a | | ab | 2f | ab | 3a | a | 3a | ab | 3a | a |
| 2f. Meningoencephalitis suspicion | bc | 2f | bc | 3b | | ab | 3a | abc | 3b | b | 2f | bc | 2c | ab |
| 3b. Sickle cell & fever | bcd | 3b | c | 2f | | ab | 2c | bc | 2f | b | 3b | cd | 3b | abc |
| 2g. Suspected pneumonia | bcde | 2e | c | 2e | | bc | 2e | bcd | 2c | bc | 2e | d | 2e | abc |
| 2c. KD on the differential | cde | 2c | c | 2c | | cd | 3b | cd | 2e | bc | 2c | d | 2f | abcd |
| 2e. Fever > 10 days | cde | 2g | d | 2g | | d | 2g | de | 2g | cd | 2g | e | 2i | abcd |
| 2i. Limp or bone/joint focus | de | 2i | d | 2b | | de | 2i | ef | 2i | de | 2i | e | 2g | bcd |
| 2h. Possible appendicitis | ef | 2b | e | 2i | | de | 2h | fg | 2h | ef | 2b | f | 2h | cd |
| 2b. Bronchiolitis suspicion | f | 2h | e | 2h | | e | 2b | g | 2b | f | 2h | f | 2b | d |

* Compact Letter Display approach: use cases are attributed a set of letters so that, when the difference in ranking between two use cases is not significant at the 5% level, they share a letter (e.g. one may be attributed the letters “ab” and the other the letters “bcd”)

**Table S3.10: Differences in rankings of adult use cases across respondent subgroups (CLD notation*)**

| **Infectious disease doctors** | | | **Other specialties** | | | **1-10 years of experience** | | | **11-20 years of experience** | | | **Over 20 years of experience** | | | **Teaching or university hospital** | | | **District or regional hospital** | | | **Other settings** | | | **Greece** | | | **Other countries** | | |
| --- | --- | --- | --- | --- | --- | --- | --- | --- | --- | --- | --- | --- | --- | --- | --- | --- | --- | --- | --- | --- | --- | --- | --- | --- | --- | --- | --- | --- | --- |
| **Use case** | **CLD** | **Use case** | | **CLD** | **Use case** | | **CLD** | **Use case** | | **CLD** | **Use case** | | **CLD** | **Use case** | | **CLD** | **Use case** | | **CLD** | **Use case** | | **CLD** | **Use case** | | **CLD** | **Use case** | | **CLD** |  |
| 4d. Immunocompromised adult | a | 4d | | a | 4d | | a | 4d | | a | 4d | | a | 4d | | ab | 4d | | a | 4d | | a | 4d | | a | 4d | | a |  |
| 4a. Fever without a focus | a | 4a | | ab | 4a | | a | 4a | | ab | 4a | | ab | 4a | | ab | 4a | | ab | 4a | | a | 4a | | ab | 4a | | a |  |
| 4c. Fever & resp. deterioration** | b | 4c | | ab | 4c | | a | 4c | | b | 4c | | bc | 4c | | b | 4c | | ab | 4b | | a | 4c | | bc | 4c | | a |  |
| 4b. Fever > 10 days | b | 4b | | bc | 4b | | a | 4b | | b | 4b | | c | 4b | | b | 4b | | abc | 4c | | ab | 4b | | c | 4b | | ab |  |
| 4f. Deteriorating elderly*** | c | 4f | | cd | 4f | | b | 4f | | c | 4f | | c | 4f | | c | 4f | | abc | 4f | | ab | 4f | | d | 4e | | bc |  |
| 4e. Atraumatic musculoskeletal^†^ | d | 4e | | d | 4e | | b | 4e | | c | 4e | | d | 4e | | c | 4e | | c | 4e | | b | 4e | | e | 4f | | c |  |

* Compact Letter Display approach: use cases are attributed a set of letters so that, when the difference in ranking between two use cases is not significant at the 5% level, they share a letter (e.g. one may be attributed the letters “ab” and the other the letters “bcd”) ** Fever and respiratory deterioration *** Elderly patient with a sudden non-specific deterioration. ^†^ Atraumatic musculoskeletal problem.

### **S3.5 Differences in overall scoring levels by subgroup**

Figure S3.1 provides average adult use case scores by clinicians working in different settings (for simplicity, we only included the two largest categories: teaching/university hospitals and district/regional hospitals on the graph). It complements Figure 2 in the main text. This graph suggests that there might also be some difference between clinicians working in different work settings, with clinicians in district/regional hospitals plausibly scoring testing somewhat higher than those in teaching/university hospitals. No other plot (comparing paediatric work setting or experience levels or adult specialties) suggested a systematic difference in overall scoring level between subgroups (we did not reproduce these plots here).

**Figure S3.1: Scores of adult use cases by work setting – boxplots with means represented by a red dot**


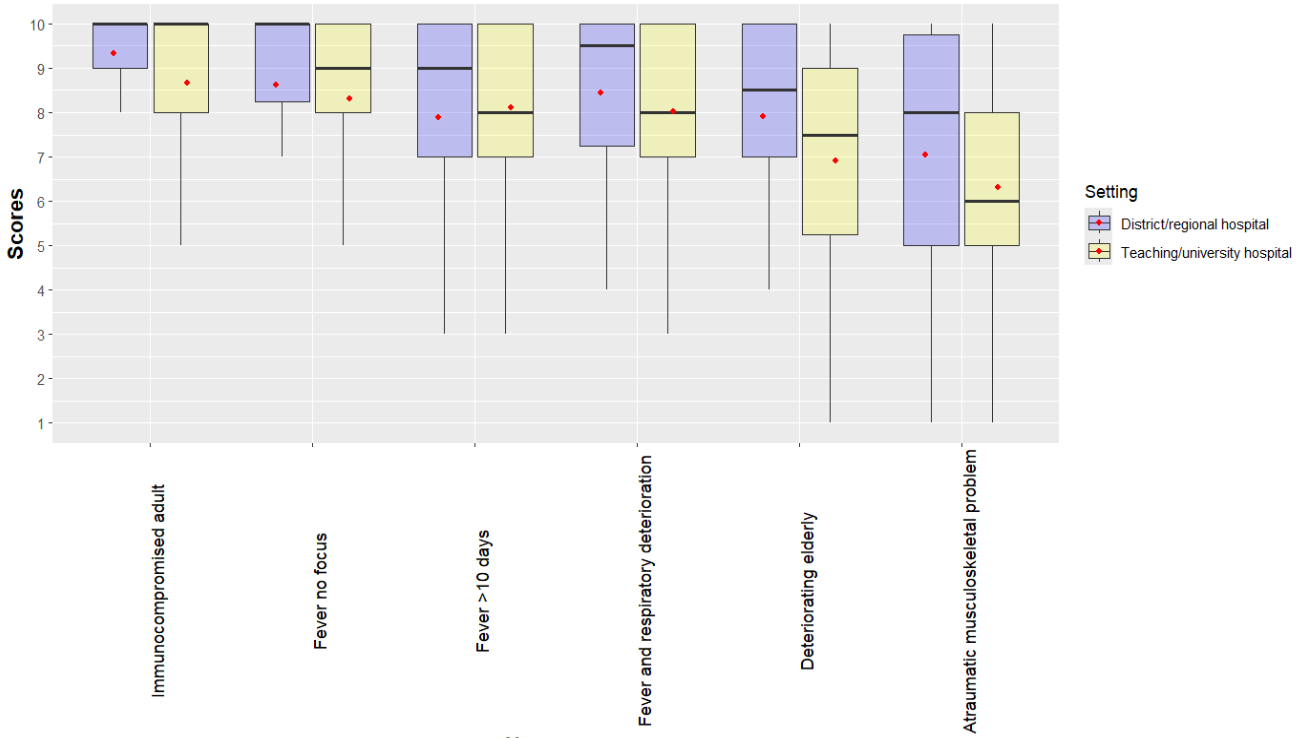


To investigate these effects quantitatively, we assumed that there may be a “respondent effect” on scores, with clinicians scoring use cases higher or lower overall because of, e.g., a greater interest in the development of new tests or a more generous scoring culture. We also assumed that the subgroup (e.g., specialty, experience, work setting or country) the respondent belongs to can influence this mean score. We finally assumed there was no interaction between the use case and subgroup variables – an assumption supported by the similarities in the relative ranking of use cases across subgroups.

We developed multi-level regression models of clinician scoring with random clinician effect and a use case dummy and clinician characteristics as independent variables. The clinician characteristics we explored were those we defined a priori and for which sample size was sufficient i.e. specialty, experience and work setting and, for adult clinicians, whether the clinician works in Greece or not, to check for the possible impact of over-representation of Greece in the adult sample on the results of our survey. We used two methods: first, ordered beta regression ^12^, a way of accounting for the metric nature of the scores, then a cumulative link mixed effects model (with logit link), which better accounts for the discrete nature of the scores.

We provide the results of the regressions for paediatric and neonatal use cases in Tables S3.11 and S3.12. These show that being an emergency clinician is associated with significant lower scores in both models, while none of the other clinician characteristics are. The Widely Applicable Information Criterion (WAIC) and Akaike information criterion (AIC) are two types of measures used to compare models based on the same datasets, with lower values being better. As shown in the tables, they do not differ much across models, but the introduction of specialty (to the exclusion of any other characteristic) leads to the lowest WAIC/AIC of all models.

The regressions for adult use cases are described in Tables S3.13 and S3.14. They suggest that clinicians with over 20 years of experience score all use cases higher than clinicians with up to 10 years of experience. Furthermore, while the coefficient for clinicians between 11 and 20 years of experience is not significant, it is also positive and a little less than half of the value of the coefficient for clinicians with over 20 years of experience, suggesting that interest for testing may increase progressively with experience. Further, the coefficients for work setting also suggest that working in a teaching/university hospital may be weakly associated with less interest in testing than working in district/regional hospitals or other work settings. Introducing experience in the model leads to a slightly higher WAIC than in the model with no clinician characteristic, whereas introducing work setting leads to the lowest WAIC of all models, though those differences are minimal, In the cumulative link mixed effects model, however, introducing experience leads to the smallest AIC, while introducing work setting (alone or alongside experience) leads to no or very minimal changes in the AIC.

**Table S3.11: Summary models: ordered beta regression of paediatric/neonatal use case scores over clinician specialty, experience and work setting** (for each parameter we give coefficients and 95% confidence intervals)

|  | **Model 0** | **Model 1** | **Model 3** | **Model 4** | **Model 5** |
| --- | --- | --- | --- | --- | --- |
| **Intercept** | 1.027 | 1.125 | 0.984 | 1.065 | 1.081 |
|  | [0.745, 1.305] | [0.823, 1.436] | [0.600, 1.381] | [0.780, 1.357] | [0.609, 1.553] |
| **Use case: neonates 3-28 days** | 0.324 | 0.317 | 0.326 | 0.321 | 0.319 |
|  | [0.062, 0.604] | [0.058, 0.582] | [0.044, 0.600] | [0.047, 0.599] | [0.037, 0.597] |
| **Use case: 1-3 months febrile infant** | 0.514 | 0.504 | 0.514 | 0.506 | 0.511 |
|  | [0.248, 0.798] | [0.241, 0.761] | [0.234, 0.794] | [0.229, 0.789] | [0.230, 0.788] |
| **Use case: bronchiolitis <18 months** | -0.959 | -0.966 | -0.957 | -0.964 | -0.962 |
|  | [-1.177, -0.703] | [-1.187, -0.725] | [-1.210, -0.708] | [-1.212, -0.716] | [-1.220, -0.719] |
| **Use case: KD on the differential** | -0.145 | -0.153 | -0.145 | -0.151 | -0.146 |
|  | [-0.370, 0.110] | [-0.383, 0.090] | [-0.398, 0.105] | [-0.405, 0.104] | [-0.409, 0.102] |
| **Use case: fever without a focus** | 0.494 | 0.483 | 0.494 | 0.487 | 0.491 |
|  | [0.224, 0.775] | [0.221, 0.742] | [0.213, 0.774] | [0.204, 0.765] | [0.211, 0.767] |
| **Use case: prolonged fever >10 days** | -0.197 | -0.210 | -0.199 | -0.208 | -0.203 |
|  | [-0.449, 0.063] | [-0.441, 0.030] | [-0.459, 0.052] | [-0.467, 0.052] | [-0.464, 0.050] |
| **Use case: meningoencephalitis suspicion** | 0.063 | 0.051 | 0.059 | 0.054 | 0.056 |
|  | [-0.181, 0.323] | [-0.194, 0.305] | [-0.209, 0.326] | [-0.212, 0.320] | [-0.210, 0.321] |
| **Use case: suspected pneumonia** | -0.452 | -0.463 | -0.455 | -0.460 | -0.458 |
|  | [-0.680, -0.206] | [-0.699, -0.227] | [-0.705, -0.205] | [-0.707, -0.212] | [-0.709, -0.209] |
| **Use case: possible appendicitis** | -1.026 | -1.043 | -1.032 | -1.038 | -1.037 |
|  | [-1.252, -0.783] | [-1.279, -0.791] | [-1.280, -0.784] | [-1.286, -0.789] | [-1.294, -0.788] |
| **Use case: atraumatic limp or bone/joint focus** | -0.562 | -0.572 | -0.557 | -0.564 | -0.561 |
|  | [-0.778, -0.323] | [-0.796, -0.332] | [-0.807, -0.312] | [-0.815, -0.318] | [-0.814, -0.316] |
| **Use case: febrile neutropenic and other immunocompromised children** | 0.324 | 0.315 | 0.322 | 0.315 | 0.320 |
|  | [0.055, 0.595] | [0.066, 0.577] | [0.049, 0.597] | [0.038, 0.587] | [0.040, 0.596] |
| **Use case: sickle cell disease and fever** | 0.000 | -0.008 | -0.001 | -0.007 | -0.003 |
|  | [-0.243, 0.270] | [-0.247, 0.238] | [-0.262, 0.259] | [-0.265, 0.252] | [-0.267, 0.254] |
| **Paediatric specialty*: emergency** |  | -0.517 |  |  | -0.575 |
|  |  | [-1.002, -0.051] |  |  | [-1.090, -0.073] |
| **Paediatric specialty*: others** |  | 0.009 |  |  | 0.085 |
|  |  | [-0.390, 0.469] |  |  | [-0.353, 0.529] |
| **Experience* (categorical): 11-20 years category** |  |  | 0.081 |  | 0.185 |
|  |  |  | [-0.381, 0.552] |  | [-0.312, 0.692] |
| **Experience* (categorical): > 20 years category** |  |  | 0.053 |  | 0.089 |
|  |  |  | [-0.404, 0.505] |  | [-0.373, 0.562] |
| **Work setting*: district/regional hospital only** |  |  |  | -0.122 | -0.189 |
|  |  |  |  | [-0.671, 0.432] | [-0.767, 0.397] |
| **Work setting*: other or multiple** |  |  |  | -0.464 | -0.584 |
|  |  |  |  | [-1.423, 0.468] | [-1.555, 0.358] |
| **Standard deviation - clinician intercept** | 1.022 | 1.004 | 1.038 | 1.035 | 1.033 |
| **WAIC**** | 805.8 | 803.6 | 805.5 | 806.2 | 805.5 |

*Reference levels: use case: <3 days old neonates; specialty: paediatric infectious disease doctors; experience: 1-10 years of experience; setting: teaching/university hospital only, ** WAIC = Widely Applicable Information Criterion (WAIC) – it is used to compare models based on the same datasets, with lower values being better.

**Table S3.12: Summary models: cumulative link mixed effects model of paediatric/neonatal use case scores over clinician specialty, experience and work setting** (for each parameter we give coefficients and 95% confidence intervals)

|  | **Model 0** | **Model 1** | **Model 3** | **Model 4** | **Model 5** |
| --- | --- | --- | --- | --- | --- |
| **Use case: neonates 3-28 days** | 0.1328 | 0.1367 | 0.1329 | 0.1308 | 0.1343 |
|  | [-0.466, 0.731] | [-0.461, 0.735] | [-0.466, 0.731] | [-0.468, 0.73] | [-0.464, 0.733] |
| **Use case: 1-3 months febrile infant** | 0.4575 | 0.4734 | 0.4571 | 0.4493 | 0.4673 |
|  | [-0.13, 1.045] | [-0.113, 1.06] | [-0.13, 1.044] | [-0.138, 1.037] | [-0.12, 1.054] |
| **Use case: bronchiolitis <18 months** | -3.0604 | -3.0466 | -3.0608 | -3.0686 | -3.0528 |
|  | [-3.646, -2.475] | [-3.631, -2.462] | [-3.646, -2.476] | [-3.654, -2.483] | [-3.638, -2.468] |
| **Use case: KD on the differential** | -1.1368 | -1.1195 | -1.1372 | -1.1448 | -1.1248 |
|  | [-1.711, -0.563] | [-1.693, -0.546] | [-1.711, -0.563] | [-1.719, -0.57] | [-1.698, -0.551] |
| **Use case: fever without a focus** | 0.4209 | 0.4351 | 0.4205 | 0.4123 | 0.4282 |
|  | [-0.166, 1.007] | [-0.151, 1.021] | [-0.166, 1.007] | [-0.175, 0.999] | [-0.158, 1.014] |
| **Use case: prolonged fever >10 days** | -1.1042 | -1.0911 | -1.1046 | -1.1123 | -1.0966 |
|  | [-1.685, -0.524] | [-1.671, -0.512] | [-1.685, -0.524] | [-1.693, -0.531] | [-1.677, -0.516] |
| **Use case: meningoencephalitis suspicion** | -0.4133 | -0.3964 | -0.4137 | -0.4218 | -0.4028 |
|  | [-1.004, 0.177] | [-0.986, 0.193] | [-1.004, 0.177] | [-1.013, 0.169] | [-0.993, 0.188] |
| **Use case: suspected pneumonia** | -1.9857 | -1.9699 | -1.9861 | -1.9936 | -1.975 |
|  | [-2.561, -1.411] | [-2.545, -1.395] | [-2.561, -1.411] | [-2.569, -1.418] | [-2.55, -1.4] |
| **Use case: possible appendicitis** | -3.3775 | -3.3669 | -3.3779 | -3.3862 | -3.3742 |
|  | [-3.982, -2.773] | [-3.971, -2.763] | [-3.982, -2.773] | [-3.991, -2.781] | [-3.979, -2.769] |
| **Use case: atraumatic limp or bone/joint focus** | -2.2084 | -2.1928 | -2.2089 | -2.2158 | -2.1968 |
|  | [-2.785, -1.632] | [-2.769, -1.617] | [-2.785, -1.633] | [-2.793, -1.639] | [-2.773, -1.62] |
| **Use case: febrile neutropenic and other immunocompromised children** | 0.1746 | 0.1893 | 0.1743 | 0.166 | 0.1824 |
|  | [-0.411, 0.76] | [-0.395, 0.774] | [-0.411, 0.76] | [-0.42, 0.752] | [-0.403, 0.768] |
| **Use case: sickle cell disease and fever** | -0.7251 | -0.711 | -0.7255 | -0.7335 | -0.7171 |
|  | [-1.301, -0.149] | [-1.286, -0.136] | [-1.301, -0.15] | [-1.31, -0.157] | [-1.293, -0.141] |
| **Paediatric specialty*: emergency** |  | -1.4199 |  |  | -1.5101 |
|  |  | [-2.327, -0.513] |  |  | [-2.433, -0.587] |
| **Paediatric specialty*: others** |  | -0.0895 |  |  | 0.0059 |
|  |  | [-0.855, 0.676] |  |  | [-0.78, 0.792] |
| **Experience* (categorical): 11-20 years category** |  |  | -0.0643 |  | 0.2186 |
|  |  |  | [-0.952, 0.824] |  | [-0.67, 1.107] |
| **Experience* (categorical): > 20 years category** |  |  | -0.044 |  | 0.051 |
|  |  |  | [-0.901, 0.813] |  | [-0.784, 0.886] |
| **Work setting*: district/regional hospital only** |  |  |  | -0.1038 | -0.2419 |
|  |  |  |  | [-1.129, 0.921] | [-1.252, 0.768] |
| **Work setting*: other or multiple** |  |  |  | -0.8278 | -1.1011 |
|  |  |  |  | [-2.58, 0.924] | [-2.812, 0.61] |
| **Standard deviation - clinician intercept** | 1.941 | 1.847 | 1.940 | 1.936 | 1.834 |
| **AIC**** | 4974.1 | 4968.1 | 4978.1 | 4977.2 | 4974.0 |

*Reference levels: use case: <3 days old neonates; specialty: paediatric infectious disease doctors; experience: 1-10 years of experience; setting: teaching/university hospital only, ** AIC = Akaike information criterion.

**Table S3.13: Summary models: ordered beta regression of adult use case scores over clinician specialty, experience, work setting and country of practice** (for each parameter we give coefficients and 95% confidence intervals)

|  | **Model 0** | **Model 1** | **Model 2** | **Model 3** | **Model 4** | **Model 5** | **Model 6** |
| --- | --- | --- | --- | --- | --- | --- | --- |
| **Intercept** | 1.333 | 1.352 | 0.992 | 1.194 | 1.418 | 0.888 | 0.973 |
|  | [1.086, 1.611] | [1.034, 1.674] | [0.593, 1.399] | [0.889, 1.519] | [1.136, 1.720] | [0.473, 1.326] | [0.481, 1.471] |
| **Use case: prolonged fever >10 days** | -0.208 | -0.215 | -0.212 | -0.214 | -0.215 | -0.215 | -0.214 |
|  | [-0.435, 0.027] | [-0.450, 0.026] | [-0.451, 0.023] | [-0.452, 0.022] | [-0.450, 0.025] | [-0.450, 0.022] | [-0.457, 0.017] |
| **Use case: fever & respiratory deterioration** | -0.240 | -0.241 | -0.242 | -0.241 | -0.242 | -0.240 | -0.241 |
|  | [-0.472, -0.001] | [-0.467, -0.006] | [-0.482, 0.000] | [-0.479, -0.006] | [-0.481, -0.008] | [-0.480, -0.008] | [-0.486, -0.004] |
| **Use case: immunocompromised adult with fever** | 0.342 | 0.341 | 0.345 | 0.343 | 0.344 | 0.344 | 0.345 |
|  | [0.092, 0.602] | [0.084, 0.612] | [0.077, 0.619] | [0.074, 0.611] | [0.082, 0.605] | [0.080, 0.616] | [0.080, 0.611] |
| **Use case: atraumatic musculoskeletal problem** | -0.878 | -0.879 | -0.877 | -0.885 | -0.882 | -0.883 | -0.885 |
|  | [-1.116, -0.650] | [-1.110, -0.647] | [-1.115, -0.646] | [-1.119, -0.656] | [-1.118, -0.655] | [-1.122, -0.654] | [-1.122, -0.654] |
| **Use case: elderly patient with a sudden non-specific deterioration** | -0.627 | -0.630 | -0.630 | -0.631 | -0.631 | -0.632 | -0.633 |
|  | [-0.850, -0.401] | [-0.861, -0.402] | [-0.864, -0.400] | [-0.866, -0.404] | [-0.867, -0.404] | [-0.868, -0.404] | [-0.868, -0.406] |
| **Specialty*: other specialties** |  | -0.022 |  |  |  |  | -0.039 |
|  |  | [-0.409, 0.375] |  |  |  |  | [-0.421, 0.353] |
| **Experience (categorical)*: 11-20 years category** |  |  | 0.295 |  |  | 0.270 | 0.271 |
|  |  |  | [-0.160, 0.759] |  |  | [-0.193, 0.740] | [-0.203, 0.732] |
| **Experience (categorical)*: > 20 years category** |  |  | 0.652 |  |  | 0.629 | 0.621 |
|  |  |  | [0.177, 1.153] |  |  | [0.139, 1.124] | [0.115, 1.131] |
| **Work setting*: district/regional hospital only** |  |  |  | 0.397 |  | 0.163 | 0.339 |
|  |  |  |  | [-0.036, 0.840] |  | [-0.352, 0.687] | [-0.128, 0.821] |
| **Work setting*: other or multiple** |  |  |  | 0.300 |  | 0.383 | 0.116 |
|  |  |  |  | [-0.221, 0.825] |  | [-0.046, 0.833] | [-0.440, 0.679] |
| **Country of practice*: not Greece** |  |  |  |  | -0.286 |  | -0.134 |
|  |  |  |  |  | [-0.703, 0.131] |  | [-0.589, 0.318] |
| **Standard deviation - clinician intercept** | 1.006 | 1.007 | 0.971 | 1.009 | 1.010 | 0.985 | 0.994 |
| **WAIC**** | 680.4 | 680.8 | 682.0 | 679.2 | 680.5 | 680.3 | 681.0 |

*Reference levels: use case: fever without a focus; specialty: paediatric infectious disease doctors; experience: 1-10 years of experience; setting: teaching/university hospital only; country: Greece. ** WAIC = Widely Applicable Information Criterion (WAIC) – it is used to compare models based on the same datasets, with lower values being better.

**Table S3.14: Summary models: cumulative link mixed effects model of adult use case scores over clinician specialty, experience, work setting and country of practice** (for each model parameter we give coefficients and 95% confidence intervals)

|  | **Model 0** | **Model 1** | **Model 2** | **Model 3** | **Model 4** | **Model 5** | **Model 6** |
| --- | --- | --- | --- | --- | --- | --- | --- |
| **Use case: prolonged fever >10 days** | -0.7193 | -0.7196 | -0.7147 | -0.7169 | -0.7205 | -0.7133 | -0.7143 |
|  | [-1.199, -0.239] | [-1.2, -0.24] | [-1.195, -0.235] | [-1.197, -0.236] | [-1.201, -0.24] | [-1.194, -0.233] | [-1.195, -0.234] |
| **Use case: fever & respiratory deterioration** | -0.5862 | -0.5863 | -0.5802 | -0.5857 | -0.5883 | -0.5806 | -0.5819 |
|  | [-1.073, -0.1] | [-1.073, -0.1] | [-1.067, -0.094] | [-1.072, -0.099] | [-1.075, -0.101] | [-1.067, -0.094] | [-1.069, -0.095] |
| **Use case: immunocompromised adult with fever** | 0.6822 | 0.6811 | 0.6865 | 0.6835 | 0.6834 | 0.6875 | 0.6867 |
|  | [0.167, 1.197] | [0.166, 1.196] | [0.171, 1.202] | [0.168, 1.199] | [0.168, 1.199] | [0.172, 1.203] | [0.172, 1.202] |
| **Use case: atraumatic musculoskeletal problem** | -2.388 | -2.3882 | -2.3786 | -2.3891 | -2.3884 | -2.3799 | -2.3803 |
|  | [-2.887, -1.889] | [-2.887, -1.889] | [-2.877, -1.88] | [-2.888, -1.89] | [-2.888, -1.889] | [-2.879, -1.881] | [-2.879, -1.881] |
| **Use case: elderly patient with a sudden non-specific deterioration** | -1.6569 | -1.6579 | -1.6517 | -1.6565 | -1.6593 | -1.6523 | -1.6546 |
|  | [-2.145, -1.168] | [-2.146, -1.169] | [-2.14, -1.164] | [-2.145, -1.168] | [-2.148, -1.171] | [-2.14, -1.164] | [-2.142, -1.167] |
| **Specialty*: other specialties** |  | -0.2255 |  |  |  |  | -0.2172 |
|  |  | [-1.009, 0.558] |  |  |  |  | [-0.973, 0.539] |
| **Experience (categorical)*: 11-20 years category** |  |  | 0.5389 |  |  | 0.4716 | 0.438 |
|  |  |  | [-0.412, 1.49] |  |  | [-0.47, 1.413] | [-0.501, 1.377] |
| **Experience (categorical)*: > 20 years category** |  |  | 1.4587 |  |  | 1.3481 | 1.3018 |
|  |  |  | [0.467, 2.45] |  |  | [0.35, 2.346] | [0.302, 2.301] |
| **Work setting*: district/regional hospital only** |  |  |  | 0.8708 |  | 0.826 | 0.734 |
|  |  |  |  | [-0.029, 1.771] |  | [-0.047, 1.699] | [-0.191, 1.659] |
| **Work setting*: other or multiple** |  |  |  | 0.9409 |  | 0.6298 | 0.5554 |
|  |  |  |  | [-0.106, 1.988] |  | [-0.406, 1.666] | [-0.528, 1.639] |
| **Country of practice*: not Greece** |  |  |  |  | -0.717 |  | -0.347 |
|  |  |  |  |  | [-1.589, 0.155] |  | [-1.249, 0.555] |
| **Standard deviation - clinician intercept** | 2.154 | 2.148 | 2.063 | 2.107 | 2.125 | 2.031 | 2.017 |
| **AIC**** | 2695.3 | 2697.0 | 2690.6 | 2694.1 | 2694.7 | 2690.6 | 2693.7 |

*Reference levels: use case: fever without a focus; specialty: paediatric infectious disease doctors; experience: 1-10 years of experience; setting: teaching/university hospital only; country: Greece** AIC = Akaike information criterion.

### **S3.6. Qualitative feedback on priorities for test development**

Clinicians provided free text responses. We expected primarily suggestions of additional use cases we may have forgotten, but clinicians were allowed to make any type of comment on the survey. Following analysis of the type of answers provided, we can classify those into five broad categories: other aetiologies they are suggesting we may focus on, priority patient profiles, priority syndromes, priority settings, and more general comments. Some of those included use cases that were already included in the survey, but that were either reiterated as being important or refined. The full results are provided in Table S3.12.

**Table S3.12: qualitative feedback from respondents on priorities for test development**

| **Category** | **Details** | **No of responses** |
| --- | --- | --- |
| Other aetiologies | Invasive fungal disease  Malignancy (to be differentiated from infection or inflammation)  Macrophage activation syndrome (children), hemophagocytic lymphohistiocytosis (adults)  Endemic zoonoses e.g., tick-borne | 2  2  2 in total  1 |
| Priority patient profile | **Profiles with impaired/suppressed immune systems:**   - Child with inborn errors of immunity - Immuno-compromised children - Adult under immune-modulating treatment - Transplant patient - Oncology patient   **Patients with an auto-immune disease**  **Complex care children or adults**  **Other profiles**   - Neonate with bronchopulmonary dysplasia - Child with severe malnutrition - Adult with a cardiac implant - Pregnant woman - Returned traveller | **5**  1  1  1  1  1  1  **2**  **5**  1  1  1  1  1 |
| Priority syndrome | **Central nervous system-related issues**:   - Meningitis/meningitis suspicion - Adult with fever and CNS symptoms or findings - Altered consciousness   **Respiratory issues/pneumonia:**   - LRTI without clear lobar pneumonia - TB vs. pneumonia - Ventilator-associated pneumonia - Pneumonia   **Shock/sepsis-related:**   - Septic shock with negative microbiology - Hypotension needing vasopressors   **Skin:**   - Skin infection with or without fever - Rash with or without fever   **Fever:**   - Refractory fever - Chronic slight fever   **Other syndromes:**   - Immuno-compromised without a fever, OD, raised inflammatory markers - Suspected infection without a fever (neonates) - > 3 weeks unexplained inflammation without a fever - Suspected bone, joint infection - Specific immunologic disease - > 60 years old adult with clinical features of urinary tract infection - Endocarditis | **5**  3  1  1  **4**  1  1  1  1  **2**  1  1  **2**  1  1  **2**  1  1  **7**  1  1  1  1  1  1  1 |
| Settings | **Specific settings mentioned**: ICU, ITU and ED after NICU discharge. | **3** |
| General comments | “In any case where a diagnostic test identifying an aetiology influences therapy i.e. antibiotic prescription, route of administration or duration, this would be helpful with most gain from high prevalence presentations / diseases or disease severity with risk in co-morbidity”  “(…) where there is a currently accepted treatment paradigm I am reasonably happy that this is less important. Where there is clinical uncertainty leading to significant overuse of antimicrobials (…) I think this would be invaluable”.  A “test may be useful if it affects management or helps to rationalise antibiotic use”.  “I think the ‘is it safe to immunosuppress/give steroids?’ question is important. (…) On ITU, I think the ‘when can we stop antibiotics?’ question is really important”. |  |

Note: certain comments may appear under multiple categories if they refer e.g. both to a specific profile of patient and a specific use case. ITU = Intensive treatment unit.

## **References**

1. Dewez JE, Pembrey L, Nijman RG, del Torso S, Grossman Z, Hadjipanayis A, et al. Availability and use of rapid diagnostic tests for the management of acute childhood infections in Europe: A cross-sectional survey of paediatricians. PLOS ONE. 2022;17(12):e0275336.

2. Eurostat. Physicians by sex and age (hlth_rs_phys). 2021.

3. Direction de la recherche dé, de l’évaluation et des statistiques (DREES),. Les internes dans les établissements de santé 2017 [Available from: <https://drees.solidarites-sante.gouv.fr/sites/default/files/2021-03/6-4.pdf>.

4. Kudryavtsev K. Moins de nouveaux internes dans les hôpitaux: les raisons d'une baisse. RTL Info. 2024.

5. NHS England. HCHS Doctors by Grade and Specialty in Trusts and CCGs - Full Time Equivalent. NHS Hospital & Community Health Service (HCHS) monthly workforce statistics2024.

6. Fédération Hospitalière de France (FHF). L'hôpital au sein de l'organisation générale de la santé 2015 [Available from: <https://www.hopital.fr/Nos-Missions/L-hopital-au-sein-de-l-organisation-generale-de-la-sante/Les-etablissements-publics-de-sante>.

7. NHS England. A&E Attendances and Emergency Admissions 2024-25 2024 [Available from: <https://www.england.nhs.uk/statistics/statistical-work-areas/ae-waiting-times-and-activity/ae-attendances-and-emergency-admissions-2024-25/>.

8. Centre national de gestion (CNG) des praticiens hospitaliers et des personnels de direction de la fonction publique hospitalière. Praticiens hospitaliers - elements statistiques sur les praticiens hospitaliers statutaires exerçant la médecine d'urgence 2017 [Available from: <https://www.cng.sante.fr/sites/default/files/media/2022-03/2017_El%C3%A9ments%20statstiques%20des%20PH%20en%20M%C3%A9decine%20d%27urgence.pdf>.

9. Brockhoff RA, Hicks SR, Salmanton-García J, Dušek D, Stahl J-P, Beeching NJ, et al. Training in infectious diseases across Europe in 2021 – a survey on training delivery, content and assessment. Clinical Microbiology and Infection. 2021;27(11):1693.e1-.e8.

10. Eisinga RA-O, Heskes T, Pelzer B, Te Grotenhuis M. Exact p-values for pairwise comparison of Friedman rank sums, with application to comparing classifiers. BMC Bioinformatics. 2017(1471-2105 (Electronic)).

11. Benjamini Y, Hochberg Y. Controlling the False Discovery Rate: A Practical and Powerful Approach to Multiple Testing. Journal of the Royal Statistical Society: Series B (Methodological). 1995;57(1):289-300.

12. Kubinec R. Ordered Beta Regression: A Parsimonious, Well-Fitting Model for Continuous Data with Lower and Upper Bounds. Political Analysis. 2023;31(4):519-36.

**Alt text Figure S3.1:** Boxplot of adult use case scores by work setting (district/regional vs. teaching/university hospitals).
