# Supplementary material for: Priorities for the development of a new rapid diagnostic test for patients with fever: a cross-sectional online survey among hospital physicians across Europe
Source: BMJ Open. 2026 Mar 24;16(3):e107663. doi: 10.1136/bmjopen-2025-107663 (PMC13034235; doi:10.1136/bmjopen-2025-107663)
Supplement: online supplemental file 3 [file bmjopen-16-3-s003.docx]

**In which patients presenting with fever do we most need a new diagnostic test? An online survey of hospital doctors across Europe**

**Journal:** European Journal of Clinical Microbiology & Infectious Diseases

**Authors**: Gabrielle Bonnet, Maryke Nielsen, Anna M. Foss, Alexandra Lewin, Ruud G. Nijman, Elizabeth Fitchett, Enitan D. Carrol, Shunmay Yeung, and the DIAMONDS consortium

**Corresponding authors:**

Gabrielle Bonnet, PhD, ORCID: 0000-0002-3539-5001

Affiliation: London School of Hygiene & Tropical Medicine

Address: Keppel Street, London, WC1E 7HT, United Kingdom

Email: [gabrielle.bonnet@lshtm.ac.uk](mailto:gabrielle.bonnet@lshtm.ac.uk)

Shunmay Yeung, PhD, ORCID: 0000-0002-0997-0850

Affiliations: London School of Hygiene & Tropical Medicine; St. Mary’s Imperial College Hospital, London

Addresses: Keppel Street, London, WC1E 7HT, United Kingdom; St Mary's Hospital, Praed St, London W2 1NY

Email: [shunmay.yeung@lshtm.ac.uk](mailto:shunmay.yeung@lshtm.ac.uk)

## **S5. DIAMONDS consortium membership**

**PARTNER: Imperial College (Coordinating Centre) (UK)**

*Chief investigator/DIAMONDS coordinator:*

Michael Levin^1^

*Principal and co-investigators (alphabetical order)^1^*

Aubrey Cunnington; Jethro Herberg; Myrsini Kaforou; Victoria J. Wright

*Section of Paediatric Infectious Diseases Research Group (alphabetical order)^1^*

Evangelos Bellos; Claire Broderick; Samuel Channon-Wells; Samantha Cooray; Tisham De (database work package lead); Giselle D’Souza; Amedine Duret; Ankita Duseja; Leire Estramiana Elorrieta; Diego Estrada-Rivadeneyra; Rachel Galassini (Clinical Trial Manager); Dominic Habgood-Coote; Shea Hamilton (Proteomics); Heather Jackson; James Kavanagh; Ilana Keren; Mahdi Moradi Marjaneh; Stephanie Menikou; Samuel Nichols; Ruud Nijman; Harsita Patel; Ivana Pennisi; Oliver Powell; Ruth Reid; Priyen Shah; Ortensia Vito; Elizabeth Whittaker; Clare Wilson; Rebecca Womersley

*Recruitment team at Imperial College Healthcare NHS Trust, London (alphabetical order)^2^*

Amina Abdulla; Sarah Darnell; Sobia Mustafa

*Engineering Team*

Pantelis Georgiou^3^ (engineering lead); Jesus Rodriguez-Manzano^4^; Nicolas Moser^3^; Ivana Pennisi^1^

^1^Section of Paediatric Infectious Disease, Imperial College London, Norfolk Place, London W2 1PG, UK

^2^Children’s Clinical Research Unit, St Mary’s Hospital, Praed Street, London W2 1NY, UK

^3^ Imperial College London, Department of Electrical and Electronic Engineering, South Kensington Campus, London, SW7 2AZ, UK

^4^ Imperial College London, Department of Infectious Disease, Section of Adult Infectious Disease, Hammersmith Campus, London, W12 0NN, UK

**UK Non-Consortium Clinical Recruiting Sites**

*Evelina London Children’s Hospital, Guy’s and St Thomas’ NHS Foundation Trust; King’s College London [combined]*

Michael Carter^1,2^ and Paul Wellman^1^; (principal investigator); Shane Tibby^1,2^ (co-investigator)

*Recruitment team (alphabetical order):* Jonathan Cohen^1^; Francesca Davis^1;^ Julia Kenny^1^;  Marie White^1^

*Laboratory team (alphabetical order):*Matthew Fish^3^; Aislinn Jennings^4^; Manu Shankar-Hari^3,4^

^1^ Evelina London Children’s Hospital, Guy’s and St Thomas’ NHS Foundation Trust, London, UK

^2^Department of Women and Children’s Health, School of Life Course Sciences, King’s College London, UK

^3^Department of Infectious Diseases, School of Immunology and Microbial Sciences, King’s College London, London, UK

^4^Department of Intensive Care Medicine, Guy’s and St Thomas’ NHS Foundation Trust, London, UK

*University Hospitals Sussex*

Katy Fidler^1^  (principal investigator); Dan Agranoff^2^ (co-investigator)

*Recruitment team*; Vivien Richmond^1,3^, Mathhew Seal^2^

^1^ Royal Alexandra Children's Hospital, University Hospitals Sussex, Brighton, UK

^2^ Dept of Infectious Diseases, University Hospitals Sussex, Brighton, UK

^3^ Research Nurse team, University Hospitals Sussex, Brighton, UK

*University Hospital Southampton NHS Foundation Trust*

Saul Faust^1^ (principal investigator); Dan Owen^1^ (co-investigator);

*Recruitment team*; Ruth Ensom^2^; Sarah McKay^2^; Diana Mondo^3^, Mariya Shaji^3^; Rachel Schranz^3^ *(alphabetical order)*

^1^ NIHR Southampton Clinical Research Facility, University Hospital Southampton NHS Foundation Trust and University of Southampton, UK

^2^ NIHR Southampton Clinical Research Facility, University Hospital Southampton NHS Foundation Trust, UK

^3^ Department of R&D, University Hospital Southampton NHS Foundation Trust, UK

*Barts Health NHS Trust*

Prita Rughani^1, 2, 3^ (principal investigator 2020-2021); Amutha Anpananthar^1, 2, 3^ (principal investigator 2021-to date); Susan Liebeschuetz^2^ (co-investigator), Anna Riddell^1^ (co-investigator)

*Recruitment team;* Divya Divakaran^3,^ Louise Han^3,^, Nosheen Khalid^1, 3,^ Ivone Lancoma-Malcolm, Jessica Schofield^3,^ Teresa Simagan^3^ *(alphabetical order)*

^1^ Royal London Hospital, Whitechapel Rd, London E1 1FR, UK

^2^ Newham University Hospital, Glen Rd, London E13 8SL, UK

^3^ Whipps Cross University Hospital, Whipps Cross Road, London, E11 1NR, UK

*Great Ormond Street Hospital for Children NHS Foundation Trust*

Mark Peters^1,2^ (principal investigator); Alasdair Bamford^1,2^ (co-investigator)

*Recruitment team;* Lauran O’Neill^1^

^1^ Great Ormond Street Hospital, London, WC1N 3JH, UK

^2^ UCL Great Ormond St Institute of Child Health, WC1N 1EH, UK

*Cambridge University Hospitals NHS Foundation Trust*

Nazima Pathan^1,2^ (principal investigator)

*Recruitment team; Esther Daubney*^1^*, Deborah White*^1^ *(alphabetical order)*

^1^Addenbrooke’s Hospital, Hills Road, Cambridge CB2 0QQ, UK

^2^Department of Paediatrics, University of Cambridge, Cambridge CB2 0QQ, UK

*University College London Hospitals NHS Foundation Trust*

Melissa Heightman^1^ (principal investigator); Sarah Eisen^1^ (co-investigator)

*Recruitment team*; Terry Segal^1^, Lucy Wellings^1^ *(alphabetical order)*

^1^ University College London Hospital, Euston Road, London NW1 2BU, UK

*St George’s University Hospitals NHS Foundation Trust*

Simon B Drysdale^1^ (principal investigator)

*Recruitment team; Nicole Branch*^1^*, Lisa Hamzah*^1^*, Heather Jarman*^1^ *(alphabetical order)*

^1^ St George’s Hospital, Blackshaw Road, London SW17 0QT, UK

*Lewisham and Greenwich NHS Trust*

Maggie Nyirenda^1, 2,^ (principal investigator)

*Recruitment team* Lisa Capozzi^1^, Emma Gardiner^1^ *(alphabetical order)*

^1^University Hospital Lewisham, London SE13 6LH, UK

^2^ Queen Elizabeth Hospital Greenwich, London SE18 4QH, UK

*Liverpool University Hospitals NHS Foundation Trust*

Robert Moots^1^ (principal investigator); Magda Nasher^2^ (principal investigator)

*Recruitment team*; Anita Hanson^2^; Michelle Linforth^1^

^1^ Aintree University Hospital, Lower Lane, Liverpool L9 7AL, UK

^2^ Royal Liverpool Hospital, Prescot St, Liverpool L7 8XP, UK

*Leeds Teaching Hospitals NHS Trust*

Sean O’Riordan^1^ (principal investigator)

*Recruitment team*; Donna Ellis^1^

^1^Leeds Children’s Hospital, Leeds LS1 3EX, UK

*King’s College Hospital NHS Foundation Trust*

Akash Deep^1^ (principal investigator)

*Recruitment team;* Ivan Caro^1^

^1^ Kings College Hospital, Denmark Hill, London SE5 9RS, UK

*Sheffield Children’s NHS Foundation Trust*

Fiona Shackley ^1^ (principal investigator);

*Recruitment team*; Arianna Bellini,^1^ Stuart Gormley^1^ *(alphabetical order)*

^1^Sheffield Children’s Hospital, Broomhall, Sheffield S10 2TH, UK

*University Hospitals of Leicester NHS Foundation Trust*

Samira Neshat^1^ (principal investigator)

^1^Leicester General Hospital, Leicester LE1 5WW, UK

*Birmingham Women’s and Children’s Hospital NHS Foundation Trust*

Barnaby J Scholefield^1^ (principal investigator)

*Recruitment team; Ceri Robbins*^1^*, Helen Winmill*^1^ *(alphabetical order)*

^1^ Birmingham Children’s Hospital, Steelhouse Lane, Birmingham B4 6NH, UK

**University of Oxford Partner**

Children’s Hospital, John Radcliffe Hospital, Oxford

Principal Investigator

Stéphane C. Paulus^1,2,3^

Co-Principal Investigator

Andrew J. Pollard^1,2,3,4^

Co-investigators

Mark Anthony^1^ (neonates)

Recruitment team

Sarah Hopton^1^, Danielle Miller^1^, Zoe Oliver^1^, Sally Beer^1^, Bryony Ward^1^

^1^John Radcliffe Hospital, Oxford University Hospitals NHS Foundation Trust, Oxford, UK

^2^Department of Paediatrics, University of Oxford, UK

^3^Oxford Vaccine Group, University of Oxford, UK

^4^NIHR Oxford Biomedical Research Centre, Oxford, UK

University of Oxford, Nepal Site

Principal Investigator

Shrijana Shrestha^1^

Co-Principal Investigator

Andrew J Pollard^2,3^

Nepal Research Team

Meeru Gurung^1^
Puja Amatya^1^

Bhishma Pokhrel^1^
Sanjeev Man Bijukchhe^1^

Madhav Chandra Gautam ^1^

Oxford Research Team

Sarah Kelly^2^
Peter O’Reilly^2^

Sonu Shrestha ^2^

^1^Paediatric Research Unit, Patan Academy of Health Sciences, Kathmandu, Nepal.

^2^Oxford Vaccine Group, Department of Paediatrics, University of Oxford, Oxford, United Kingdom.

^3^NIHR Oxford Biomedical Research Centre, Oxford, United Kingdom.

**SERGAS Partner (Spain)**

Principal Investigators

Federico Martinón-Torres^1^

Antonio Salas^1,2^

GENVIP RESEARCH GROUP (in alphabetical order):

Fernando Álvez González^1^, Sonia Ares Gómez^1^, Xabier Bello^1,2^, Mirian Ben García^1^, Fernando Caamaño Viña^1^, Sandra Carnota^1^, María José Curras-Tuala^1,2^, Ana Dacosta Urbieta^1^, Carlos Durán Suárez^1^, Isabel Ferreiros Vidal^1^, Luisa García Vicente^1^, Alberto Gómez-Carballa^1,2^, Jose Gómez Rial^1^, Pilar Leboráns Iglesias^1^, Narmeen Mallah^1^, Federico Martinón-Torres^1^, Nazareth Martinón-Torres^1^, José María Martinón Sánchez^1^, Belén Mosquera Pérez^1^, Jacobo Pardo-Seco^1,2^, Sara Pischedda^1,2^, Sara Rey Vázquez^1^, Irene Rivero Calle^1^, Carmen Rodríguez-Tenreiro^1^, Lorenzo Redondo-Collazo^1^, Antonio Salas^1,2^, Sonia Serén Fernández^1^, Marisol Vilas Iglesias^1^.

^1^ Translational Pediatrics and Infectious Diseases, Pediatrics Department, Hospital Clínico Universitario de Santiago, Santiago de Compostela, Spain, and GENVIP Research Group (www.genvip.org), Instituto de Investigación Sanitaria de Santiago, Universidad de Santiago de Compostela, Galicia, Spain.

^2^ Unidade de Xenética, Departamento de Anatomía Patolóxica e Ciencias Forenses, Instituto de Ciencias Forenses, Facultade de Medicina, Universidade de Santiago de Compostela, and GenPop Research Group, Instituto de Investigaciones Sanitarias (IDIS), Hospital Clínico Universitario de Santiago, Galicia, Spain

^3^ Fundación Pública Galega de Medicina Xenómica, Servizo Galego de Saúde (SERGAS), Instituto de Investigaciones Sanitarias (IDIS), and Grupo de Medicina Xenómica, Centro de Investigación Biomédica en Red de Enfermedades Raras (CIBERER), Universidade de Santiago de Compostela (USC), Santiago de Compostela, Spain

**Liverpool Partner**

Principal Investigators

Enitan D Carrol^1,2,^

Research Group (in alphabetical order):

Elizabeth Cocklin^1^, Rebecca Beckley^1,2,3^, Abbey Bracken^1^, Ceri Evans^1,2^, Aakash Khanijau^1^, Rebecca Lenihan^1^, Nadia Lewis-Burke^1^, Karen Newall^3^, Sam Romaine^1^, Jennifer Whitbread ^3^

^1^ Department of Clinical Infection, Microbiology and Immunology, University of Liverpool Institute of Infection, Veterinary and Ecological Sciences , Liverpool, England

^2^ Alder Hey Children’s Hospital, Department of Infectious Diseases, Eaton Road, Liverpool, L12 2AP

^3^Alder Hey Children’s Hospital, Clinical Research Business Unit, Eaton Road, Liverpool, L12 2AP

**NATIONAL AND KAPODISTRIAN UNIVERSITY OF ATHENS (Greece)**

Principal Investigator: Maria Tsolia^1^

Co-Investigator: Irini Eleftheriou^1^

PID Unit: Nikos Spyridis^1^, Maria Tambouratzi^1^

Pediatric Rheumatology Unit: Despoina Maritsi^1^

Lab: Antonios Marmarinos^1^, Marietta Xagorari^1^

Recruitment teams:

Adult COVID19- Infectious Diseases: Lourida Panagiota, Pefanis Aggelos^2^

Adult COVID19: Akinosoglou Karolina, Gogos Charalambos, Maragos Markos^3^

Adult Inflammatory Diseases-Oncology: Voulgarelis Michalis , Stergiou Ioanna^4^

^1^2^nd^ Department of Pediatrics, National and Kapodistrian University of Athens (NKUA), Children’s Hospital “P, and A. Kyriakou”, Athens, Greece

^2^1^st^ Department of Infectious Diseases, General Hospital “Sotiria”

^3^Pathology Department, University of Patras, General Hospital “Panagia i Voithia”

^4^Pathophysiology Department, Medical Faculty, National and Kapodistrian University of Athens (NKUA), General Hospital “Laiko”

**Newcastle upon Tyne Hospitals NHS Foundation Trust and Newcastle University (UK) combined**

Principal Investigator:

Marieke Emonts ^1,2,3^ (all activities)

Co-investigators

Emma Lim^2,3,6^ (all activities)

John Isaacs^1^ (adult inflammatory)

Recruitment team (alphabetical), datamanagers, and GNCH Research unit:

Kathryn Bell^4^, Stephen Crulley^4^, Daniel Fabian^4^, Evelyn Thomson^4^, Diane Wallia^4^, Caroline Miller^4^  , Ashley Bell^4^

PhD Students/medical staff DIAMONDS

Fabian J.S. van der Velden^1,2^ (all activities), Geoff Shenton^7^ (oncology), Ashley Price^8,9^ (Adult COVID)

Students

Owen Treloar ^1,2^ (quality control, data management and analysis)

Daisy Thomas^1,2^ (recruitment)

Author Affiliations:

^1^ Translational and Clinical Research Institute, Newcastle University, Newcastle upon Tyne UK

^2^Great North Children’s Hospital, Paediatric Immunology, Infectious Diseases & Allergy, Newcastle upon Tyne Hospitals NHS Foundation Trust, Newcastle upon Tyne, United Kingdom.

^3^NIHR Newcastle Biomedical Research Centre based at Newcastle upon Tyne Hospitals NHS Trust and Newcastle University, Westgate Rd, Newcastle upon Tyne NE4 5PL, United Kingdom

^4^Great North Children’s Hospital, Research Unit, Newcastle upon Tyne Hospitals NHS Foundation Trust, Newcastle upon Tyne, United Kingdom.

^6^Population Health Sciences Institute, Newcastle University, Newcastle upon Tyne, UK

^7^Great North Children’s Hospital, Paediatric Oncology, Newcastle upon Tyne Hospitals NHS Foundation Trust, Newcastle upon Tyne, United Kingdom.

^8^Department of Infection & Tropical Medicine, Newcastle upon Tyne Hospitals NHS Foundation Trust, Newcastle upon Tyne, United Kingdom

^9^NIHR Newcastle In Vitro Diagnostics Co-operative (Newcastle MIC), Newcastle upon Tyne, United Kingdom.

**Servicio Madrileño de Salud (SERMAS) - Fundación Biomédica del Hospital Universitario 12 de Octubre (FIB-H12O) (Spain)**

Principal Investigators

Pablo Rojo^1,3^

Cristina Epalza ^1,2^

SERMAS/FIB-H120 team:

Serena Villaverde^1^, Sonia Márquez^2^, Manuel Gijón ^1,2^, Romina Varchetta ^2^, Fátima Machín^2^, Laura Cabello^2^, Irene Hernández^2^, Lourdes Gutiérrez^2^, Ángela Manzanares ^1,2^

Author Affiliations:

^1^ Servicio Madrileño de Salud (SERMAS),Pediatric Infectious Diseases Unit, Department of Pediatrics, Hospital Universitario 12 de Octubre, Madrid, Spain

^2^Fundación Biomédica del Hospital Universitario 12 de Octubre (FIB-H12O), Unidad Pediátrica de Investigación y Ensayos Clínicos (UPIC), Hospital Universitario 12 de Octubre, Instituto de Investigación Sanitaria Hospital 12 de Octubre (i+12), Madrid, Spain.

^3^ Universidad Complutense de Madrid, Faculty of Medicine, Department of Pediatrics, Madrid, Spain.

**Amsterdam University Medical Center (Amsterdam UMC), University of Amsterdam**

Principal Investigator:

T.W. (Taco) Kuijpers MD PhD ^1,2^ (all activities)

Co-investigators

M. (Martijn) van de Kuip MD PhD ^1^ (infectious disease)

A.M. (Marceline) van Furth MD PhD ^1^ (infectious disease)

J.M. (Merlijn) van den Berg MD PhD ^1^ (inflammatory disease)

Hospital Team (all activities):

Giske Biesbroek MD PhD ^1^, Floris Verkuil MD (PhD student) ^1^, Carlijn (C.W.) van der Zee MD (start 1/8/2022, PhD student) ^1^

Recruitment:

Dasja Pajkrt MD PhD ^1^, Michael Boele van Hensbroek MD PhD ^1^, Dieneke Schonenberg MD ^1^, Mariken Gruppen MD ^1^, Sietse Nagelkerke MD PhD ^1,2^, medical students

Laboratory Team:

Machiel H Jansen ^1^, Ines Goetschalckx (PhD student) ^2^

Author Affiliations:

^1^ Amsterdam UMC, Emma Children's Hospital, Dept of Pediatric Immunology, Rheumatology and Infectious Disease, University of Amsterdam, The Netherlands

^2^ Sanquin, Dept of Molecular Hematology, University Medical Center, Amsterdam, The Netherlands

**Bambino Gesù Children’s Hospital (Rome-Italy)**

Principal Investigator

Lorenza Romani 1

Maia De Luca 1

Recruitment Team

Sara Chiurchiù 1

Costanza Tripiciano 1

Stefania Mercadante 1

Affiliation

1 Infectious Disease Unit, Academic Department of Pediatrics, Bambino Gesù Children's Hospital, IRCCS, Rome 00165, Italy

**ERASMUS MC-Sophia Children’s Hospital**

*Principal Investigator*

Clementien L. Vermont²

*Research group*

Henriëtte A. Moll¹, Dorine M. Borensztajn¹, Nienke N. Hagedoorn, Chantal Tan ¹, Joany Zachariasse ¹, Medical students ¹

Additional investigator

W Dik ^3^

¹ Erasmus MC-Sophia Children’s Hospital, Department of General Paediatrics, Rotterdam, the Netherlands

² Erasmus MC-Sophia Children’s Hospital, Department of Paediatric Infectious Diseases & Immunology, Rotterdam, the Netherlands

^3^ Erasmus MC, Department of immunology, Rotterdam, the Netherlands

**TAIWAN**

Ching-Fen, Shen

Department of Pediatrics, National Cheng Kung University Hospital, College of Medicine, National Cheng Kung University

Tainan, Taiwan

**Riga Stradins University (Riga, Latvia)**

Principal Investigator:

Dace Zavadska ^1,2^ (all activities)

Co-investigators

Sniedze Laivacuma ^1,3^ (adult cohorts)

Recruitment team:

Aleksandra Rudzate ^1,2^, Diana Stoldere ^1,2^, Arta Barzdina ^1,2^, Elza Barzdina ^1,2^, Sniedze Laivacuma^1,3^, Monta Madelane ^1,3^

Laboratory

Dagne Gravele ^2^, Dace Svile^2^

Author Affiliations:

^1^ Riga Stradins University, Riga, Latvia

^2^ Children clinical university hospital, Riga, Latvia

^3^ Riga East clinical university hospital, Riga, Latvia

**Assistance Publique - Hôpitaux de Paris**

Principal Investigator:

Romain Basmaci ^1,2^

Co-investigator:

Noémie Lachaume ^1^

Recruitment team:

Pauline Bories ^1^, Raja Ben Tkhayat ^1^, Laura Chériaux ^1^, Juraté Davoust ^1^, Kim-Thanh Ong ^1^, Marie Cotillon ^1^, Thibault de Groc ^1^, Sébastien Le ^1^, Nathalie Vergnault ^1^, Hélène Sée ^1^, Laure Cohen ^1^, Alice de Tugny ^1^, Nevena Danekova ^1^

Author Affiliations:

^1^ Service de Pédiatrie-Urgences, AP-HP, Hôpital Louis-Mourier, F-92700 Colombes, France

² Université Paris Cité, Inserm, IAME, F-75018 Paris, France

**BioMérieux**

Principal Investigator:

Marine Mommert-Tripon

Co-investigator:

Karen Brengel-Pesce

Author Affiliations:

bioMérieux - Open Innovation & Partnerships Department, Lyon, France

University Medical Centre Ljubljana, Slovenia

Principal Investigator: Marko Pokorn ^1,2,3^

Co-Investigator: Mojca Kolnik^2^

Research Group (in alphabetical order):

Tadej Avčin^2,3^, Tanja Avramoska^2^, Natalija Bahovec^1^, Petra Bogovič^1^, Lidija Kitanovski^2,3^, Mirijam Nahtigal^1^, Lea Papst^1^, Tina Plankar Srovin^1^, Franc Strle^1,2^, Katarina Vincek^1^.

Affiliations:

1. Department of Infectious diseases, University Medical Centre Ljubljana, Slovenia
2. University Children's Hospital, University Medical Centre Ljubljana, Slovenia
3. Faculty of Medicine, University of Ljubljana, Slovenia
4. Centre for Clinical research, University Medical Centre Ljubljana

**University Medical Center Utrecht, Utrecht, The Netherlands**

Principal Investigator

Michiel van der Flier^1,5 (^Pediatric Infectious Diseases and Immunology)

Co-investigators

Wim J.E. Tissing^5^ (Pediatric Oncology)

Roelie M. Wösten-van Asperen^2^ (Pediatric Intensive Care Unit)

Sebastiaan J Vastert^3^ (Pediatric Rheumatology)

Daniel C Vijlbrief^4^ (Pediatric Neonatal Intensive Care)

Louis J. Bont^1,5 (^Pediatric Infectious Diseases and Immunology)

PhD student

Coco R. Beudeker^1,5 (^Pediatric Infectious Diseases and Immunology)

Affiliations:

1.Pediatric Infectious Diseases and Immunology, 2. Pediatric Intensive Care Unit 3. Pediatric Rheumatology 4. Pediatric Neonatal Intensive Care, Wilhelmina Children’s Hospital, University Medical Center Utrecht, Utrecht, The Netherlands

5. Princess Maxima Center for Pediatric Oncology, Utrecht, The Netherlands

**University of Bern Partner**

**Inselspital, Bern University Hospital, University of Bern, Switzerland**

Principal Investigator

Philipp Agyeman^1^

Co-Investigators

Christoph Aebi^1^, Nina Schöbi^1^

Recruitment team

Mariama Usman^1^, Stefanie Schlüchter^1^

^1^ Department of Pediatrics, Inselspital, Bern University Hospital, University of Bern, Switzerland

**University of Zürich Partner**

**Kinderspital Zürich, University Children’s Hospital Zurich**

Principal Investigator

Luregn Schlapbach^1,2^

Co-Investigators

Cornelia Hagmann^1^, Florian Zapf^1^, Philipp Baumann^1^, Barbara Brotschi^1^

Recruitment team

Elisa Zimmermann^1^ PhD, Marion Meier^1^, Kathrin Weber^1^

^1^ Department of Intensive Care and Neonatology, and Children`s Research Center, University Children`s Hospital Zurich, Zurich, Switzerland

^2^ Child Health Research Centre, The University of Queensland, Brisbane, Australia

Micropathology Ltd

Micropathology Ltd, The Venture Center, University of Warwick Science Park, Sir William Lyons Road, Coventry, CV4 7EZ

Principle Investigator; Prof Colin Fink

Co Investigators: Marie Voice, Leo Calvo-Bado, Michael Steele, Jennifer Holden, Andrew Taylor, Ronan Calvez

Research group: Catherine Davies, Benjamin Evans, Jake Stevens, Peter Matthews, Kyle Billing

**Medical University of Graz, Austria (MUG)**

Principal Investigator:

Werner Zenz^1^ (all activities)

Co-investigators (in alphabetical order):

Alexander Binder^1^ (grant application)

Benno Kohlmaier^1^ (study design, recruitment)

Daniela S. Kohlfürst^1^ (study design)

Nina A. Schweintzger^1^ (all activities)

Christoph Zurl^1^ (study design, recruitment)

Recruitment team, data managers, laboratory work (in alphabetical order):

Susanne Hösele^1^, Piyush G. Gampawar^1^, Barbara Kapo^1^, Manuel Leitner^1^, Lena Pölz^1^, Alexandra Rusu^1^, Glorija Rajic^1^, Bianca Stoiser^1^, Martina Strempfl^1,^ Manfred G. Sagmeister^1^

Clinical recruitment partners (in alphabetical order):

Sebastian Bauchinger^1^, Martin Benesch^3^, Astrid Ceolotto^1^, Ernst Eber^2^, Siegfried Gallistl^1^, Harald Haidl^1^, Almuthe Hauer^1^, Christa Hude^1^, Andreas Kapper^7^, Markus Keldorfer^5^, Sabine Löffler^5^, Tobias Niedrist^6^, Heidemarie Pilch^5^, Andreas Pfleger^2^, Klaus Pfurtscheller^4^, Siegfried Rödl^4^, Andrea Skrabl-Baumgartner^1^, Volker Strenger^3^, Elmar Wallner^7^

Author Affiliations:

^1^ Department of Pediatrics and Adolescent Medicine, Division of General Pediatrics, Medical University of Graz, Graz, Austria

^2^Department of Pediatric Pulmonology, Medical University of Graz, Graz, Austria

^3^Department of Pediatric Hematooncology, Medical University of Graz, Graz, Austria

^4^Paediatric Intensive Care Unit, Medical University of Graz, Graz, Austria

^5^University Clinic of Pediatrics and Adolescent Medicine Graz, Medical University Graz, Graz, Austria

^6^Clinical Institute of Medical and Chemical Laboratory Diagnostics, Medical University Graz, Graz, Austria

^7^Department of Internal Medicine, State Hospital Graz II, Location West, Graz, Austria

**Project partner BBMRI-ERIC**

Maike K. Tauchert

Author affiliation:

Biobanking and BioMolecular Resources Research Infrastructure - European Research Infrastructure Consortium (BBMRI-ERIC), Neue Stiftingtalstrasse 2/B/6, 8010, Graz, Austria

**LMU Munich Partner (Germany)**

Principal Investigator:

Ulrich von Both^1,2^ MD, FRCPCH (all activities)

Research group:

Laura Kolberg¹ MSc (all activities)

Patricia Schmied¹ (Study physician), Ioanna Mavridi¹ (PhD student), Irene Alba-Alejandre^3^ MD (Study physician)

Clinical recruitment partners (in alphabetical order):

Katharina Danhauser, MD^6^, Nikolaus Haas, MD^11^, Florian Hoffmann, MD^10^, Matthias Griese, MD^7^, Tobias Feuchtinger, MD^5^, Sabrina Juranek, MD^4^, Matthias Kappler, MD^7^, Eberhard Lurz, MD^8^, Esther Maier, MD^4^, Karl Reiter, MD^10^, Carola Schoen, MD^10^, Sebastian Schroepf, MD^9^

Author Affiliations:

¹ Division of Pediatric Infectious Diseases, Department of Pediatrics, Dr. von Hauner Children’s Hospital, University Hospital, LMU Munich, Munich, Germany

^2^ German Center for Infection Research (DZIF), Partner Site Munich, Munich, Germany

^3^ Department of Gynecology and Obstetrics, University Hospital, LMU Munich, Munich, Germany

^4^ Division of General Pediatrics, Department of Pediatrics, Dr. von Hauner Children’s Hospital, University Hospital, LMU Munich, Munich, Germany

^5^ Division of Pediatric Haematology & Oncology, Department of Pediatrics, Dr. von Hauner Children’s Hospital, University Hospital, LMU Munich, Munich, Germany

^6^ Division of Pediatric Rheumatology, Department of Pediatrics, Dr. von Hauner Children’s Hospital, University Hospital, LMU Munich, Munich, Germany

^7^ Division of Pediatric Pulmonology, Department of Pediatrics, Dr. von Hauner Children’s Hospital, University Hospital, LMU Munich, Munich, Germany

^8^ Division of Pediatric Gastroenterology, Department of Pediatrics, Dr. von Hauner Children’s Hospital, University Hospital, LMU Munich, Munich, Germany

^9^ Neonatal Intensive Care Unit, Department of Pediatrics, Dr. von Hauner Children’s Hospital, University Hospital, LMU Munich, Munich, Germany

^10^ Paediatric Intensive Care Unit, Department of Pediatrics, Dr. von Hauner Children’s Hospital, University Hospital, LMU Munich, Munich, Germany

^11^ Department of Pediatric Cardiology and Pediatric Intensive Care, University Hospital, LMU Munich, Germany

**London School of Hygiene and Tropical Medicine (LSHTM)**

Principal Investigator, WP7 lead and SP9 co-lead: Shunmay Yeung ^1,2,3^

Research group:

Gabrielle Bonnet ^1^ (Economic evaluation and health systems, modelling, pilot demonstration)

Maryke Nielsen ^1^ (Pilot demonstration, clinical, economic evaluation and health systems)

Elizabeth Fitchett^1^ (Clinical, evaluation)

Manuel Dewez ^1^ (Economics and health systems)

David Bath^3^ (Economics and health systems, modelling)

1. Clinical Research Department, Faculty of Infectious and Tropical Disease, London School of Hygiene and Tropical Medicine, London
2. Department of Paediatrics, St. Mary’s Imperial College Hospital, London
3. Department of Global Health and Development, Faculty of Public Health and Policy, London School of Hygiene and Tropical Medicine, London

**Medical Research Council Unit The Gambia at LSHTM**

P O Box 273,

Fajara, The Gambia

Site Principal Investigator

Effua Usuf

Additional Investigators

Kalifa Bojang (Co Investigator)

Anna Roca (Co Investigator)

Isatou Sarr (Senior Scientist)

Momodou Saidykhan (Nurse Co-ordinator)

Ebrahim Ndure (Data Manager)

Affiliations

Medical Research Council at LSHTM, Fajara

**European Bioinformatics Institute (EMBL-EBI), United Kingdom**

*Co-investigators*

Pedro Madrigal (Bioinformatics) ¹

Silvie Fexova (Data curation) ¹

*Affiliation:*

¹ EMBL-EBI, Wellcome Genome Campus, Hinxton, Cambridgeshire, CB10 1SD, UK

**Department of Pediatric Infectious Diseases, Medical University of Bialystok, Poland**

Site Principal Investigator

Artur Sulik^1^

Co-Investigators:

Kacper Toczylowski^1^

Dawid Lewandowski^1^

^1^Department of Pediatric Infectious Diseases, Medical University of Bialystok, Poland
