# Supplementary material for: Priorities for the development of a new rapid diagnostic test for patients with fever: a cross-sectional online survey among hospital physicians across Europe
Source: BMJ Open. 2026 Mar 24;16(3):e107663. doi: 10.1136/bmjopen-2025-107663 (PMC13034235; doi:10.1136/bmjopen-2025-107663)
Supplement: Supplementary file 4 [file bmjopen-2025-107663.reviewer_comments.pdf]

PEER REVIEW HISTORY

BMJ Open publishes all reviews undertaken for accepted manuscripts. Reviewers are asked to complete a checklist review form and are provided with free text boxes to elaborate on their assessment. These free text comments are reproduced below.

ARTICLE DETAILS

Title (Provisional)

Priorities for the development of a new rapid diagnostic test for febrile patients: a cross-sectional online survey among hospital physicians across Europe

Authors

Bonnet, Gabrielle; Nielsen, Maryke Joanne; Foss, Anna M; Lewin, Alex; Nijman, Ruud Gerard; Fitchett, Elizabeth; Carrol, Enitan; Yeung, Shunmay; DIAMONDS consortium, on behalf of the

---

VERSION 1 - REVIEW

**Reviewer**	**1**
**Name**	**Krishnan, Yamini**
**Affiliation**	**MVR Cancer Centre and Research Institute**
**Date**	**09-Sep-2025**
**COI**	**None**

The study describes in detail on the design and development process. The authors have highlighted the strengths and limitations of their analysis. The supplementary materials also highlight the detailing that has gone into the study

Minor comment

1.The authors have not mentioned whether an Ethical clearance has been taken and the IEC number

2. The survey has a question on whether the respondents are aware of the DIAMONDS project. It would be interesting to know how many of the respondents are part of the project or was it an exclusion criteria. Was this discussed with the stastical

3. There is no specific mention of any exclusion criteria

4.Table 1 - Labelling of the diagram is not clear and needs to be corrected

---

**Reviewer**	**2**
**Name**	**Baymakova, Magdalena**

**Affiliation**	**Military Medical Academy**
**Date**	**15-Dec-2025**
**COI**	**None**

Comments to the Authors:

I read this manuscript "In Which Patients Presenting with Fever do We Most Need a New Diagnostic Test? An Online Survey of Hospital Doctors across Europe" (Manuscript ID: bmjopen-2025-107663) submitted to a peer-reviewed journal "BMJ Open" (Impact Factor: 2.3; CiteScore: 4.5). The idea of the manuscript is good. However, the manuscript needs Major Revision.

TITLE: I recommend a new title as follows – "Priorities for the development of a new rapid diagnostic test for febrile patients: a cross-sectional online survey among hospital physicians across Europe".

ABSTRACT: I have no comments or recommendations for this section.

INTRODUCTION: I have no comments or recommendations for this section.

MATERIALS & METHODS:

(1) I recommend that the authors add a new subsection " Inclusion & Exclusion Criteria" (3-4-5 sentences).

(2) I recommend that authors add to the new subsection "Ethical Considerations" (3-4 sentences). Authors should add information about Number and Date of the Protocol with which the Local Ethics Committee authorized the present research. Authors should add information about Written Informed Consent obtained and signed by each participant in the current survey.

(3) I recommend that the authors add a new Figure "Flow chart showing the selection process of research participants".

RESULTS: I have no comments or recommendations for this section.

DISCUSSION: I recommend that the authors add a new subsection "Limitations of the Study" (3-4 sentences).

REFERENCES: I have no comments or recommendations for this section.

TABLES: I have no comments or recommendations for this section.

FIGURES: See recommendations for section "Materials & Methods".

**Reviewer**	**3**
**Name**	**Chen Lie, Khie**

Affiliation **Universitas Indonesia Center for Clinical Epidemiology and Evidence-Based Medicine, Internal Medicine**

Date **20-Feb-2026**

COI **None**

Thank you for submitting manuscript to BMJ Open.

There are some comment and questions regarding your manuscript:

1. Your study explores the needs and priority of attending Physician for new diagnostic test that are not informed the characterized the type of examination whether to assess the inflammatory of etiological diagnostic, and benefit through the 'generic' existing test in term of speed to obtain the results, easy to performed and lower cost.

2. Questionnaire are provided closed answers that tend to direct respondent choose the provided answers. The qualitative free feedback from respondents, showed there are wide range of other answers that has not accommodate in the questionnaires.

3. Range of respondents invite in this study are heterogenous, approximately half are infectious diseases or immunologist pediatrics and adults Physician, that have different medical problems, type etiological pathogens and setting practice compared to others.

4. Dissemination of questionnaire are trough the medical professional organizations, that could potentially selection bias, as the Physician invited could have the same interest.

Method how to select the inviting respondents, how many respondents are invited, and rate of responds are not informed in the manuscript.

5. Although wide coverage of the study including 30 European countries, mostly adults Physician respondents are from Greek, that may not represent all European countries. Geographical variation including different infectious diseases etiological pattern including antimicrobial resistance in Greek are different from others European countries.

VERSION 1 - AUTHOR RESPONSE

Reviewer: 1
Dr. Yamini Krishnan, MVR Cancer Centre and Research Institute
Comments to the Author:

The study describes in detail on the design and development process. The authors have highlighted the strengths and limitations of their analysis. The supplementary materials also highlight the detailing that has gone into the study

Thank you very much for reviewing our paper!

Minor comment
1.The authors have not mentioned whether an Ethical clearance has been taken and the IEC number

Thank you for this comment. The ethical approval details (LSHTM 30182) were included in the original submission, but the way we submitted it means it may or may not have appeared within the paper you reviewed. We have now added a statement about ethics and consent under an "Ethical considerations" subsection at the end of the Methods section to ensure it is more prominent. This section reads:

"This research was approved by the London School for Hygiene and Tropical Medicine's ethics committee on 27 March 2024 (Reference number 30182). Written informed consent was obtained electronically from all participants before they were given access to the survey."

2. The survey has a question on whether the respondents are aware of the DIAMONDS project. It would be interesting to know how many of the respondents are part of the project or was it an exclusion criteria. Was this discussed with the statistical

We thank the reviewer for this pertinent question. The awareness question was originally included as a communication impact metric for the DIAMONDS consortium to evaluate its outreach efforts. Regarding potential bias, there was broad awareness of DIAMONDS as 29% of respondents had heard of the project. However, this was primarily through professional society presentations or peer-reviewed literature. A granular analysis of the responses revealed that only a negligible minority (<2%, n < 5) identified as members of the project, mostly as clinicians at participating hospitals who were not involved in the diagnostic test design. Because this group represents such a tiny fraction of the total sample, they were not excluded, nor were they analysed as a separate subgroup, as their inclusion is statistically insufficient to shift the consensus or the relative rank order of the use cases. We have added a brief clarification to the Results (Respondents section) regarding this independent clinical consensus: "*while 29% of respondents were aware of the DIAMONDS consortium through professional outreach, direct involvement was negligible (<2%). This small minority was included in the primary analysis, which reflects a broadly independent clinical consensus*."

3. There is no specific mention of any exclusion criteria

Thank you very much for this comment. We have now added a section in methods, just under "target population", entitled "Inclusion and exclusion criteria" that says: "*Respondents were included if they were doctors working in a hospital in Europe (including non-EU countries) and involved in patient assessment and diagnosis. Exclusion criteria were: 1) not being a doctor (for example, an allied health professional) 2) not working in a hospital, 3) working outside of Europe, 4) not being involved in patient assessment and diagnosis, 5) not consenting to the survey. These could be assessed through answers to the survey*."

4.Table 1 - Labelling of the diagram is not clear and needs to be corrected

We have clarified the labels by providing full clinical descriptions in the legend and S1 Appendix, and we have increased the final image resolution to ensure all text is legible in the typeset version.

Here is the revised title and description of Fig 2 (formerly Fig 1)

"Fig 2: Clinician priority ratings for 20 clinical use cases

(A) Distribution of respondent scores for each use case, represented as box-and-whisker plots. (B) Mean priority scores with associated 95% confidence intervals.

Use case age-group is indicated by background shading: blue for neonatal, green for paediatric, and pink for adult presentations. In Panel A, black dots represent the mean score, horizontal lines indicate the median, boxes represent the interquartile range (IQR), and whiskers show the full data range. In Panel B, error bars represent 95% confidence intervals obtained through the adjusted bootstrap percentile method.. Use case descriptions on the x-axis are abbreviated; full clinical descriptions are provided in S1 Appendix."

Reviewer: 2

Dr. Magdalena Baymakova, Military Medical Academy

Comments to the Authors:

I read this manuscript "In Which Patients Presenting with Fever do We Most Need a New Diagnostic Test? An Online Survey of Hospital Doctors across Europe" (Manuscript ID: bmjopen-2025-107663) submitted to a peer-reviewed journal "BMJ Open" (Impact Factor: 2.3; CiteScore: 4.5). The idea of the manuscript is good. However, the manuscript needs Major Revision.

Thank you very much for reviewing this manuscript! We share our responses to your comments and suggestions and how we have incorporated them in the revised manuscript below.

TITLE: I recommend a new title as follows – "Priorities for the development of a new rapid diagnostic test for febrile patients: a cross-sectional online survey among hospital physicians across Europe".

Thank you for the suggestion, we have made the correction.

ABSTRACT: I have no comments or recommendations for this section.

INTRODUCTION: I have no comments or recommendations for this section.

MATERIALS & METHODS:

I recommend that the authors add a new subsection " Inclusion & Exclusion Criteria" (3-4-5 sentences).

Thank you for noting. We have now added a section in methods, just under "target population", entitled "Inclusion and exclusion criteria" that says: "*Respondents were included if they were doctors working in a hospital in Europe (including non-EU countries) and involved in patient assessment and diagnosis. Exclusion criteria were: 1) not being a doctor (for example, an allied health professional) 2) not working in a hospital, 3) working outside of Europe, 4) not being involved in patient assessment and diagnosis, 5) not consenting to the survey. These could be assessed through answers to the survey*."

(2) I recommend that authors add to the new subsection "Ethical Considerations" (3-4 sentences). Authors should add information about Number and Date of the Protocol with which the Local Ethics Committee authorized the present research. Authors should add information about Written Informed Consent obtained and signed by each participant in the current survey.

Thank you for this comment. The ethical approval details (LSHTM 30182) were included in the original submission, but the way we submitted it means it may or may not have appeared within the paper you reviewed. We have now added a statement about ethics and consent under an "Ethical considerations" subsection at the end of the Methods section to ensure it is more prominent. This section reads:

"This research was approved by the London School for Hygiene and Tropical Medicine's ethics committee on 27 March 2024 (Reference number 30182). Written informed consent was obtained electronically from all participants before they were given access to the survey."

(3) I recommend that the authors add a new Figure "Flow chart showing the selection process of research participants".

We thank you for this suggestion. We have added a new Fig 1 in the "respondents" subsection of the "results" section, which illustrates the participant flow from initial landing page access to final inclusion. As this was an 'open' survey disseminated via third-party professional societies, we have followed the CHERRIES guidelines by documenting the numbers reaching the landing page, those excluded via our screening questions/consent process, and the final analytical sample.

Please note that with the addition of the Flow Chart as Fig 1, all subsequent figures in the manuscript have been re-numbered accordingly.

RESULTS: I have no comments or recommendations for this section.

DISCUSSION: I recommend that the authors add a new subsection "Limitations of the Study" (3-4 sentences).

We thank you for this suggestion. In line with this and other reviewer comments, we have restructured the Discussion to include a dedicated "Strengths and limitations" subsection within the "Discussion" section. We have expanded this part of the manuscript significantly to address specific concerns regarding the sampling frame, the over-representation of certain subgroups, and geographical generalisability.

REFERENCES: I have no comments or recommendations for this section.

TABLES: I have no comments or recommendations for this section.

FIGURES: See recommendations for section "Materials & Methods".

Done, as explained above.

<u>Reviewer: 3</u>

Dr. Khie Chen Lie, Universitas Indonesia Center for Clinical Epidemiology and Evidence-Based Medicine

Comments to the Author:

Thank you for submitting manuscript to BMJ Open.

Thank you very much for agreeing to review your paper and providing many thoughtful comments!

There are some comment and questions regarding your manuscript:

1. Your study explores the needs and priority of attending Physician for new diagnostic test that are not informed the characterized the type of examination whether to assess the inflammatory of etiological diagnostic, and benefit through the 'generic' existing test in term of speed to obtain the results, easy to performed and lower cost.

Thank you very much for this technical observation. We agree that the performance characteristics (speed, cost, ease of use) are critical for the eventual adoption of any new diagnostic. However, we would like to clarify that this survey was conducted during the discovery stage when the test was not yet fully finalized and its primary objective was to identify clinical needs (prioritising patient "use cases" and aetiologies) to inform the development of the test and associated analyses.

In this context, the 'Information Section' provided to all respondents informed them that the survey concerned 'new host biomarker-based tests.' In the 'Use Case' section, we explicitly listed the potential aetiologies (viral, bacterial, inflammatory, etc.) as seen in Part 2 and 3 of our survey. Because the discovery work was ongoing (e.g., exploring the feasibility of differentiating Gram+ from Gram-), we intentionally did not provide fixed parameters for speed or cost. This was to avoid biasing clinicians toward existing 'generic' tests and instead capture their 'ideal' requirements for a multi-aetiology tool.

While this study identifies the clinical demand, the eventual success of the DIAMONDS test will depend on its ability to outperform current 'generic' standards in terms of turnaround time and cost-effectiveness. Assessing test capabilities and potential cost-effectiveness is part of ongoing work which is going to be reported upon in additional papers. We have added a sentence to the methods section, study design subsection, acknowledging that:

" To prioritise clinical need over specific technical specifications during the discovery phase of the test, this survey employed a technology-agnostic design. Respondents were informed the focus was on 'host biomarker-based' and 'multi-aetiology' tests; however, specific parameters for speed and cost were omitted to capture unbiased clinician priorities."

2. Questionnaire are provided closed answers that tend to direct respondent choose the provided answers. The qualitative free feedback from respondents, showed there are wide range of other answers that has not accommodate in the questionnaires.

We thank you for highlighting the value of the qualitative feedback. We agree that these responses provide important information, including identification of specific aetiologies or syndromes of interest such as fungal diseases or macrophage activation syndrome. We would like to clarify, though, that free-text suggestions were provided by only 14% of the total sample, which is in line with usual percentages of free text comments in clinician surveys. Furthermore, within the free text comments and suggestions, many were suggestions that provided additional nuances to the existing use cases rather than proposing new categories. In this context, we feel that our pre-defined use cases achieved good "content validity" (i.e. they appear to have captured the primary concerns of the respondents), but agree that our article would have benefited from greater clarity regarding these free text comments. Accordingly, we have slightly revised the results section, as follows:

" Free-text suggestions (provided by 14% of respondents) focused primarily on settings, patient profiles, aetiologies and syndromes. Many of the suggestions provided additional nuance to an existing use case. Additional aetiologies/causes of illness suggested for inclusion on a test included: fungal, malignancy, macrophage activation syndrome, and endemic zoonoses. Comments highlighted the importance of immune-compromised patients as a target population. Full results are in S3.6 appendix."

3. Range of respondents invite in this study are heterogenous, approximately half are infectious diseases or immunologist pediatrics and adults Physician, that have different medical problems, type etiological pathogens and setting practice compared to others.

Yes, we agree. The heterogeneity of the respondent pool was an intentional feature of our study design, intended to reflect the real-world multidisciplinary nature of patient assessment and diagnosis in European hospitals.

To ensure this diversity did not compromise the validity of our findings by asking clinicians to rank use cases they do not usually see in their practice, we specifically asked clinicians to identify the age groups they treat on a routine basis, following which clinicians were only shown this/these specific age group(s). In addition, within the age range clinicians were shown, every use case included an 'I do not see this type of patient' option. The low frequency of this selection suggests that respondents felt clinically competent to prioritize the cases presented. Most importantly, we conducted a sensitivity analysis to determine if priorities differed by specialty. Note that, there, we distinguished between absolute ratings (how high a score was given) and relative ranking (the order of priority). While we noted some variation in 'enthusiasm' (e.g., more experienced clinicians giving slightly higher average scores to adult cases), the relative ranking of use cases remained consistent across all subgroups, suggesting a robust consensus that transcends specific medical specialties.

We have added a sentence to the Discussion to more strongly highlight that the consensus remained consistent despite the diverse clinical backgrounds of the participants "*The relative rank order of the use cases remained stable across all subgroups, indicating a broad consensus on clinical priorities regardless of the respondent's specific background. Conversely, absolute scores varied slightly by specialty and experience level.*"

4. Dissemination of questionnaire are through the medical professional organizations, that could potentially selection bias, as the Physician invited could have the same interest. Method how to select the inviting respondents, how many respondents are invited, and rate of responds are not informed in the manuscript.

We thank you for the opportunity to clarify our sampling methodology. We have added the following sentence "*The survey was disseminated through the newsletters and listservs of professional societies and networks including….*" , and revised the sub-heading of the section which was previously labelled "*Dissemination*" to "*Participant recruitment*" .

With regards selection bias, we agree that using professional societies excludes clinicians who are not members of such organisations. However, this is the established pragmatic method for reaching a Europe-wide clinical audience such as the one required for this study. To mitigate internal bias, we disseminated the survey through multiple, distinct professional societies (Emergency, Infectious Diseases, Paediatrics…) to ensure a broad range of perspectives. We agree this still leaves gaps in the respondent population that should be acknowledged: we accordingly added the following in the discussion section

"to address one of the main limitations of the study in relation to our approach to participant selection through professional societies, and the potential bias introduced by the over-representation of certain group. As clinicians' choices are likely to be influenced by their specialty and where they work we performed extensive analysis as to look for systematic differences and were reassured to find limited differences in the ranking of use cases and in aetiology preferences across different specialties, and in comparing between Greek and non-Greek respondents. While clinical priorities did appear consistent across our sample, we acknowledge that local variations in infectious disease epidemiology and antimicrobial resistance (AMR) patterns may influence the eventual real-world utility of a new test in different European regions."

Regarding the sampling frame and response rate, due to the nature of dissemination via third-party society newsletters and listservs, the total number of individuals who viewed the invitation (the denominator) cannot be precisely determined. This is because membership lists often overlap (clinicians belonging to multiple societies) and societies do not always share the exact number of active 'opens' for their newsletters. Instead, we have followed the CHERRIES guidelines for reporting web-based surveys.

We already highlighted the numbers reaching the information and consent page (270 participants), proportions not consenting (0.7%), consenting but not in target group (0.7%) or having left all answers empty despite starting the survey (0.4%). What was missing was the proportion of respondents who did not respond to or said they did not see a specific use case despite routinely seeing the corresponding age group. This is reported in a new appendix (S3.2.1. Non-response among clinicians seeing a specific age range).

All but one of these percentages were below 5% and most were below 2%. The exception relates to "Unwell neonate – early onset". This is explained by the fact that, among our respondents who said they routinely worked with neonates, there was a substantial proportion who were not neonatologists but paediatricians: these would more often see older neonates who have left the neonatal unit then are brought back to care as they become sick than the youngest neonates who often have never left the neonatal units. We have referred to that appendix in the main text (results section, respondents subsection): "*Response rates for individual use cases are in S3.2.1 Appendix*."

We have also added a statement in the discussion section acknowledging that while our 'open' recruitment method is standard for European-wide clinician research, it does not allow for a traditional response rate calculation. "*Finally, because the survey was disseminated as an 'open' survey via professional medical societies, the total number of clinicians reached via these various channels cannot be precisely quantified (due to overlapping memberships and third-party distribution), hence a traditional response rate could not be calculated".*

5. Although wide coverage of the study including 30 European countries, mostly adults Physician respondents are from Greek, that may not represent all European countries. Geographical variation including different infectious diseases etiological pattern including antimicrobial resistance in Greek are different from others European countries.

We thank you for this important observation. We agree that the high proportion of adult respondents from Greece (74% of the adult sample) is a limitation, particularly given the specific infectious disease and AMR patterns in that region.

As noted in the manuscript, we made extensive efforts to recruit adult clinicians via multiple European networks; however, recruitment was most successful through Greek professional societies. To address the potential for geographical bias, we conducted a sensitivity analysis comparing the responses of Greek adult clinicians to those from the rest of Europe. We found no evidence that clinical priorities or use-case rankings differed significantly between these groups. We have explicitly stated in the Discussion that generalisability to the broader European adult population is limited. To further address your point regarding local context, we have updated the Discussion to specifically acknowledge that while clinical priorities appear consistent in our sample, local etiological and AMR patterns must be considered when implementing such tests in different European settings:

"As clinicians' choices are likely to be influenced by their specialty and where they work we performed extensive analysis as to look for systematic differences and were reassured to find limited differences

in the ranking of use cases and in aetiology preferences across different specialties, and in comparing between Greek and non-Greek respondents. While clinical priorities did appear consistent across our sample, we acknowledge that local variations in infectious disease epidemiology and antimicrobial resistance (AMR) patterns may influence the eventual real-world utility of a new test in different European regions."
